# Monitoring Astrocytic Proteome Dynamics by Cell Type-Specific Protein Labeling

**DOI:** 10.1371/journal.pone.0145451

**Published:** 2015-12-21

**Authors:** Anke Müller, Anne Stellmacher, Christine E. Freitag, Peter Landgraf, Daniela C. Dieterich

**Affiliations:** 1 Neural Plasticity and Communication, Institute for Pharmacology and Toxicology, Otto–von–Guericke–University Magdeburg, Magdeburg, Germany; 2 Research Group Neuralomics, Leibniz Institute for Neurobiology, Magdeburg, Germany; 3 Center for Behavioral Brain Sciences, Magdeburg, Germany; University of Muenster, GERMANY

## Abstract

The ability of the nervous system to undergo long-term plasticity is based on changes in cellular and synaptic proteomes. While many studies have explored dynamic alterations in neuronal proteomes during plasticity, there has been less attention paid to the astrocytic counterpart. Indeed, progress in identifying cell type-specific proteomes is limited owing to technical difficulties. Here, we present a cell type-specific metabolic tagging technique for a mammalian coculture model based on the bioorthogonal amino acid azidonorleucine and the mutated *Mus musculus* methionyl-tRNA synthetase^L274G^ enabling azidonorleucine introduction into *de novo* synthesized proteins. Azidonorleucine incorporation resulted in cell type-specific protein labeling and retained neuronal or astrocytic cell viability. Furthermore, we were able to label astrocytic *de novo* synthesized proteins and identified both Connexin-43 and 60S ribosomal protein L10a upregulated upon treatment with Brain-derived neurotrophic factor in astrocytes of a neuron-glia coculture. Taken together, we demonstrate the successful dissociation of astrocytic from neuronal proteomes by cell type-specific metabolic labeling offering new possibilities for the analyses of cell type-specific proteome dynamics.

## Introduction

Dynamic adaptations in synaptic strength are thought to be the basis for information processing and memory consolidation in neuronal circuits. In order to fully understand how different modulatory inputs result in plasticity, another major cell type of the central nervous system has to be taken into account, the astrocyte. The intensive crosstalk between neurons and astrocytes at synapses led to the concept of the tripartite synapse model that now incorporates modulatory influences on synaptic transmission emanating from astrocytes [1].

Given the close partnership between neurons and astrocytes, it is clear that adaptive mechanisms must also exist in the astrocytic part of the tripartite synapse. Recent reports prove that this is indeed the case. Astrocytic glutamate receptors were found to be altered in mouse hippocampal slices after long-term activity changes [2]. In addition, adaptive mechanisms, based on astrocytic process dynamics, have been observed in the hypothalamus of lactating animals [3]. Also, the observed mid- to long-term persistent changes of astrocytic calcium signaling strongly suggest the existence of long-term adaptive mechanisms in astrocytes [4–6].

Such long-term adaptations to changes of synaptic transmission, as has been described in neurons, include alterations in proteome composition resulting partly from the induction of gene expression and subsequent protein translation events [7,8]. Up to now, however, little is known whether similar activity-induced proteome plasticity occurs in astrocytes. This is due, in part, to the lack of appropriate techniques that can access specific changes in the astrocytic proteome, distinct from neuronal proteome changes. The first evidences for astrocytic protein expression dynamics involve the assessment of candidate proteins, for example the transcriptional regulator Nrf2, the glutamate transporter Glt1 and the secreted protein SPARC [9–11]. Candidate-based approaches, however, cannot be applied to top-down investigations or to an analysis of the regulation of ubiquitously expressed proteins, owing to the technical difficulty associated with separating neuronal and astrocytic proteomes. State of the art methods to separate astrocytic and neuronal transcriptomes or proteomes rely on transgenic mouse lines that use cell type-specific promoters of GFAP, S100-B or Aldh1l1 to overexpress reporter proteins exclusively in astrocytes, respectively [12–14]. However, a dynamic change in the proteome composition upon neuronal activity is difficult to investigate applying these techniques. Although easier to approach experimentally, the use of astrocytic monocultures or astrocytic cell lines as alternative model systems are limited in their applicability, not only because of the missing neuronal dialog partner but also because of the lack of physiological comparability to astrocytes *in vivo*. Critically, direct contact with neurons in culture is thought to reduce the reactive status of astrocytes and to modulate cellular characteristics, as revealed by extensive gene expression changes, pointing towards the tight interdependency of both cell types [15]. In order to allow for an analysis of protein synthesis dynamics in astrocytes that directly contact neurons, we developed a metabolic labeling technique that is cell type-specific and allows us to monitor dynamic changes of the astrocytic proteome within a heterogeneous cellular model system.

Our technology is based on the bioorthogonal non-canonical amino acid azidonorleucine (ANL), used as a surrogate for methionine (Met) in protein translation. It is built on well-described labeling technologies utilizing azidohomoalanine (AHA) as applied in several different systems [16–20]. Integration of either AHA or ANL into proteins brings along an azide group that allows conjugation to either a fluorescent or biotin tag by an azide-alkyne cycloaddition reaction, commonly referred to as a 'click reaction' [21]. This enables one to track *de novo* synthesized proteins within cells or to apply affinity purification of tagged proteins followed by subsequent identification via mass spectrometry [16,22].

Application of the long-chained amino acid ANL is combined with the expression of a mutated methionyl-tRNA synthetase (MetRS) enabling the activation of ANL and the subsequent coupling to the correspondent tRNA^Met^ due to an enlarged substrate binding pocket [23,24]. Successful cell type-specific labeling with ANL was previously demonstrated by Ngo *et al*. who site-specifically replaced the initiator-methionine residue of proteins with ANL in the presence of the *Escherichia coli* MetRS mutant NLL-EcMetRS [25]. Very recently, a cell type-specific labeling approach with another enzyme variant MetRS^L262G^ demonstrated the successful and specific ANL integration into proteins of diverse cell types of living *Drosophila melanogaster*, as well as the discovery of a global neuronal protein translation reduction in a *Drosophila* model of Charcot-Marie-Tooth disease [26,27]. Meanwhile another attempt to substitute the amino acid phenylalanine by a bioorthogonal analogue proved to differentially label cell type-specific proteomes in *Caenorhabditis elegans* [28].

Here, we applied for the first time ANL and the murine MetRS^L274G^ in a cell type-specific labeling approach in a mammalian cell culture model. We examined three mutant versions of the murine MetRS (mMetRS) and identified mMetRS^L274G^ as a potent activator for ANL in cell culture. The cell type-specific expression of EGFP-mMetRS^L274G^ in astrocytes only enabled the differentiation of astrocytic proteins from neuronal proteins in a neuron-glia coculture system. Furthermore, we applied the technique to identify changes in astrocytic protein expression in response to Brain-derived neurotrophic factor (BDNF) treatment. By purifying cell type-specific labeled proteins and by defining precise labeling times we contribute new insights into astrocytic protein dynamics in the context of neuronal activity.

## Material and Methods

### Antibodies and reagents

All reagents were ACS grade and purchased from Sigma-Aldrich unless noted otherwise.

For immunoblotting the following primary antibodies were used: rabbit polyclonal anti-Biotin (1:10000, A150-109A, Bethyl Laboratories Inc.), rabbit polyclonal anti-Ubiquitin (1:2000, ADI-SPA-200, Enzo Life Sciences); rabbit polyclonal anti-Connexin-43 (1:5000, ab11370, Abcam), mouse monoclonal anti-Rpl10a (1:1000, ab55544, Abcam), chicken polyclonal anti-GFAP (1:10000, ab4674, Abcam), rabbit polyclonal anti-Fibronectin (1:1000, F3648, Sigma-Aldrich). Respective peroxidase conjugated secondary antibodies raised against mouse, rabbit or chicken primary antibodies (1:7500, all from Jackson ImmunoResearch Laboratories) were applied.

For immunocytochemistry stainings mouse monoclonal or rabbit polyclonal antibodies against MAP2 (1:500, M1406, Sigma-Aldrich and 1:1000, ab32454, Abcam) chicken polyclonal anti-GFAP (1:1000, ab4674, Abcam), rabbit polyclonal anti-GFP (1:2000, ab290, Abcam), rabbit polyclonal anti-Connexin-43 (1:500, ab11370, Abcam), mouse monoclonal anti-Rpl10a (1:250, ab55544, Abcam), mouse monoclonal anti-GM130 (1:1000, 610822, BD Biosciences) and guinea pig anti-Synaptophysin (1:2000, 101004, Synaptic Systems) were applied. For the analysis of altered protein expression in cocultured astrocytes rabbit polyclonal antibody anti-Fibronectin (1:500, F3648, Sigma-Aldrich) was used. Dye-conjugated secondary antibodies from goat or donkey (1:2000, Jackson ImmunoResearch Laboratories or Invitrogen) were applied for visualization by fluorescence microscopy.

### Generation of EGFP-mMetRS^L274G^ and lentiviral expression vectors

The Met binding pocket of the mouse methionyl-tRNA synthetase (mMetRS; BC079643.1) was enlarged by introducing a point mutation leading to an exchange of glycine to leucine at amino acid position 274 as described in S1 File–Supplemental Material and Methods.

For the generation of lentiviral constructs the FUGW expression vector was used [29]. For morphological analysis the expression of EGFP-mMetRS^L274G^ was driven from the ubiquitin promoter of FUGW. For cell type-specific expression of EGFP or EGFP-mMetRS^L274G^ the ubiquitin promoter of the lentiviral FUGW expression vector was replaced by the GFAP promoter version gfaABC_1_D [30]. Cloning of both constructs is described in S1 File–Supplemental Material and Methods.

Lentivirus preparation is described in S1 File–Supplemental Material and Methods. For all experiments rat cortical neuron-glia cocultures and astrocytic monocultures were infected four days prior use.

### Animals

Cultures were obtained from E18–19 or P2–P3 rats (Wistar). Adult rats were housed in groups of 5–6 animals, at constant temperature (22±2°C) and relative humidity (50%) under a regular 12 h light-dark schedule (lights on 6 AM–6 PM) with food and water available *ad libitum*. Adult rats were deeply anaesthetized with Isofluran Baxter (Baxter Deutschland GmbH) prior decapitation using an animal guillotine. Embryos and pubs were decapitated without prior treatment using decapitation scissors. In the present experiments, animal care and procedures were approved and conducted under established standards of the German federal state of Sachsen-Anhalt (Institutional Animal Care and Use Committee: Landesverwaltungsamt Sachsen-Anhalt; License No. 42505-2-1172 UniMD Germany in accordance with the European Communities Council Directive; 86/609/EEC). Any effort was made to minimize the number of animals used and their suffering.

### Metabolic labeling and cell manipulation

*Rattus norvegicus* glia monocultures and neuron-glia cocultures were prepared and cultured as described in S1 File–Supplemental Material and Methods. Labeling experiments were done in Met-free medium supplemented with 4 mM Met, AHA or ANL as described in more detail in S1 File–Supplemental Material and Methods (see also S1 File–Supplemental Material and Methods for preparation of AHA and ANL).

For the determination of cell vitality, neuron-glia cocultures, infected with LVEGFP-mMetRS^L274G^, were incubated with either 4 mM Met, AHA or ANL for 4h and dead cells were further live-stained with propidium iodide as described in S1 File–Supplemental Material and Methods. In BDNF experiments either 50 ng/ml BDNF (Alomone Labs) or 2 μM TTX (Tocris) were applied together with 4 mM AHA or ANL in Met-free medium and further processed for fluorescent or bioorthogonal non-canonical amino acid tagging (FUNCAT or BONCAT). For the quantification of Cx43 and Rpl10a in EGFP-positive astrocytes, 50 ng/ml BDNF were applied to the standard culture medium for indicated times and cells were fixed and processed for immunocytochemistry. Glia monocultures were labeled in Met-free DMEM as described for HEK293T cells in S1 File–Supplemental Material and Methods and incubated with 4 mM AHA. Both treatment and incubation times were done as described for neuron-glia cocultures. For the analysis of translation rates, neuron-glia cocultures, infected with the lentivirus LVEGFP-mMetRS^L274G^, were labeled with 4 mM ANL at DIV 22 for 0 h, 1 h, 2 h and 4 h and further processed for FUNCAT and immunocytochemistry as described below.

### Bioorthogonal non-canonical amino acid tagging (BONCAT)

Cell pellets of HEK293T cells or neuron-glia cocultures were lysed in 1x PBS (pH 7.8)supplemented with 0.1% SDS, 0.2% Triton X-100, 250 U/ml Benzonase® nuclease, 1x cOmplete™ EDTA-free protease inhibitor cocktail (Roche) and ‘click reaction’ was performed as described previously with minor adjustments [31]. Briefly, the following reactants were added to lysed samples: 0.2 mM Tris[(1-benzyl-1*H*-1,2,3-triazol-4-yl)methyl]amine (TBTA), 25 μM biotin-alkyne tag (biotin-PEO-propagylamid; see S1 File–Supplemental Material and Methods), 0.2 mg/ml copper(I)bromide-suspension (Acros) and cell extracts were incubated over night (ON) under permanent agitation at 4°C. Precipitates were removed by centrifugation at 3000 g for 5 min after reaction and protein lysates were further processed for immunoblotting (see S1 File–Supplemental Material and Methods) or affinity purification as described below.

### Met competition assay

For the analysis of ANL integration in the presence of Met, cells overexpressing EGFP-mMetRS^L274G^ from pEGFP-C1 (Clontech) were incubated both in Met-free culture medium as well as in standard HEK293T cell culture medium that has an intrinsic Met concentration of 0.2 mM (Invitrogen). Cells were supplemented with either 4 mM AHA, 4 mM Met or 4 mM ANL for 1 h as described in detail above.

### Fluorescent non-canonical amino acid tagging (FUNCAT)

The FUNCAT reaction for either fixed HEK293T cells, fixed glia monocultures or fixed neuron-glia cocultures was performed as described previously using the Tetramethylrhodamine (TAMRA)-alkyne tag (Invitrogen) at a final concentration of 0.2 μM [32].

### Immunocytochemistry and microscopy

Immunofluorescence stainings were performed as follows: cells were fixed with 4% paraformaldehyde (Roth) in 1x PBS (pH 7.4) and subsequently blocked with B-Block solution (10% horse serum, 5% sucrose, 2% bovine serum albumin, 0.2% Triton^TM^ X-100 in 1x PBS, pH 7.4) for 1.5 h at room temperature (RT) or directly used for first antibody incubation after FUNCAT reaction. First and secondary antibodies were diluted in B-Block solution as listed above and incubated for 2 h (primary antibody) and 1 h (secondary antibody) at RT each followed by three washing steps using 1x PBS (pH 7.4). Cover slips were mounted in 10% mowiol 4–88 (Calbiochem), 25% glycerol and 2.5% DABCO in Tris-HCl buffer (pH 7.8). Bis-Benzimide (Hoechst 33258, Invitrogen) was used at a concentration of 5 μg/ml in the second of three washing steps with PBS (pH 7.4) after secondary antibody incubation. Immunocytochemistry stainings were analyzed by fluorescence microscopy. For the analysis of newly synthesized proteins via FUNCAT, pictures were taken at an Axio observer.Z1 microscope. For FISH experiments and for the immunocytochemistry stainings of S5 Fig, Z-stacks were generated at an Axio observer.Z1 microscope with an LSM 710 confocal unit (both Carl Zeiss AG).

### Quantification of signal intensities

For the quantification of newly synthesized proteins in astrocytes EGFP was expressed via lentiviral transduction of LVGFAPEGFP and additionally enhanced with immunocytochemical stainings in combination with stainings against GFAP to confirm astroglial origin of the cell. Image acquisition was performed with identical exposure times and settings for all experimental groups applying fluorescence microscopy. Image processing and analysis was done using ImageJ (National Institutes of Health; USA; http://rsb.info.nih.gov/ij/index.html).

For the determination of TAMRA intensities upon BDNF application, the background was subtracted for each fluorescence channel. The EGFP channel was used to generate a mask utilizing the threshold function in ImageJ and mean signal intensities of TAMRA were determined within the borders of the mask. Experiments that were included in the statistical analysis showed also an effect in neurons upon BDNF treatment as described previously [22]. Statistical analysis was performed using One-way ANOVA test with about 15–20 cells of each 2–3 coverslips per experimental group. For the quantification of Rpl10a a mask using the cell type-specific EGFP expression was generated as described above and mean signal intensities for Rpl10a stainings were determined within the mask. To quantify newly synthesized Cx43, signal intensities of Cx43 staining were measured within the Golgi apparatus using an antibody for the Golgi marker GM130 (BD Biosciences) to generate a mask. Statistical analysis was performed for Rpl10a and Cx43 quantification using a student's t-test with 10–15 cells of each 1–2 coverslips per experimental group (80–130 cells in total per experimental condition). For the analysis of ongoing translation in neuronal dendrites MAP2-positive dendrites were straightened using the ImageJ plug-in Straighten and a section of 20–40 μm starting from the most proximal part of the dendrite was used for analysis. A mask was generated utilizing the threshold function in ImageJ based on the MAP2 staining and the TAMRA intensity was measured within the mask. Background staining measured at 0 h was subtracted from values for all time points for each experiment. Mean fluorescence intensities were summarized from three independent experiments comprising 2–3 cover slips per experimental group. For the quantification of FISH signals, positive probe signals within the EGFP mask were counted in Z-stacks with the 3D object counter plugin of Fiji software (http://fiji.sc/Fiji). Statistical analysis was done utilizing a student’s t-test with a total number of 60 to 72 cells per experimental condition out of three independent experiments.

### Affinity purification of biotin-tagged proteins

For the isolation of biotin-tagged proteins after BONCAT via affinity-purification, samples were desalted using PD-10 Desalting Columns (GE Healthcare) into 1x PBS (pH 7.8) supplemented with 0.05% SDS, 1 x cOmplete™ EDTA-free protease inhibitor cocktail (Roche) as well as 1% NP40 and incubated with High Capacity NeutrAvidin^TM^ Agarose-Resin (Thermo Scientific) ON at 4°C.

The agarose was washed once with 1% SDS in 1x PBS (pH 7.8), four times with 1x PBS (pH 7.8) supplemented with 0.05% SDS and 1% NP40 and three times with 1x PBS (pH 7.8) for 10 min each. Bound proteins were eluted by incubating the samples at 95°C for 5 min in 4x SDS sample buffer and analyzed using immunoblotting (see S1 File–Supplemental Material and Methods).

### Fluorescence *in situ* hybridization

Dissociated cortical neurons were infected with LVGFAPEGFP, treated with 50 ng/ml BDNF for 1 h at DIV 22 and fixed with a lysine-phosphate-buffered 4% paraformaldehyde solution supplemented with 1.35% glucose, 0.1 M lysine-HCl and 0.01 M sodium metaperiodate (pH7.4) for 30 min at RT. *In situ* hybridization was performed using the QuantiGene® ViewRNA ISH Cell Assay kit (eBioscience) as described previously [33] with probes binding to following target regions: rat GFAP: 232–1171 nt (NM_017009); rat Cx43: 93–1116 nt (NM_012567). Proteinase K treatment was omitted to preserve the integrity of proteins. After hybridization steps cells were washed 3 times with 1x PBS (pH 7.4) and blocked with 10% horse serum, 2% BSA, 5% sucrose and 0.2 mg/ml Saponin in 1x PBS (pH 7.4) for 1 h. Subsequently, cells were incubated with the primary antibody diluted in the blocking solution for 1 h and further processed using immunocytochemistry as described above.

## Results

### *De novo* astrocytic protein translation is altered by BDNF in astrocytes of primary cocultures

Dynamic adaptation of the proteome to changes in the cellular environment is one of the universal hallmarks for cellular function and is the basis for a successful collaboration of cells within a complex heterogeneous cellular system. This holds true also for the nervous system, where long-term adaptation of neuronal synapses depends on protein synthesis and a new set of proteins might support the stabilization of potentiated synapses [34]. The presence of similar proteome changes as response to activity alterations in the astroglial part of the tripartite synapse is not known so far and would ultimately require an adaptation to altered neuronal activity on the basis of astrocytic protein expression. To analyze protein translation events solely in astrocytes that are tightly connected with neurons is technically extremely challenging, since state of the art proteomic approaches demand a separation of both cells to differentiate between their proteomes. This is generally either achieved by mono-cultivation of specific cells or by cell-separating approaches, both interrupting more or less cellular contact and communication of both cell types necessary for a physiological function.

**Fig 1:**
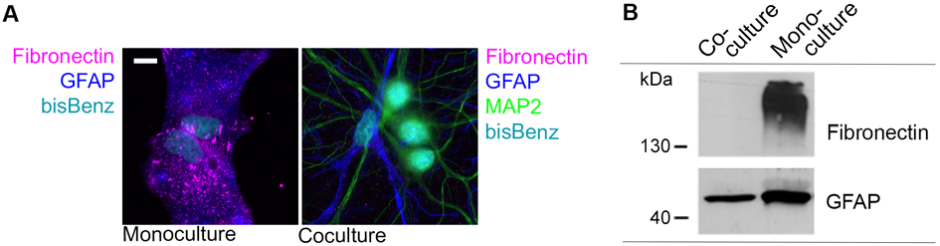
Astrocytes lose fibroblast-like morphology in a neuron-glia coculture system. (A) Astrocytes (DIV 22) grown in monoculture exhibit a fibroblast-like morphology and express Fibronectin, as illustrated by immunocytochemistry. Direct contact with neurons in coculture (DIV 22) led to increased process formation resembling a morphology more similar to protoplasmic astrocytes, and a drastically reduced expression of Fibronectin. Nuclei are stained with bis-Benzimide (bisBenz) and astrocytes or neurons are identified by either GFAP or MAP2 immunostainings, respectively (scale bar = 10 μm). (B) Immunoblot analysis of lysates derived from astrocytic monocultures and neuron-glia cocultures (DIV 22) demonstrate a loss of Fibronectin expression and a reduction in GFAP in cocultures.

In order to understand the dynamics of the astrocytic proteome for neuron-glia interaction and, thus, ultimately for general brain function, we started to use rat neuron-glia cocultures that allow direct interaction of astrocytes and neurons as well as an easy access to monitor ongoing proteome changes within the heterogeneous cellular system. Astrocytes that are able to directly contact neurons obtain a stellate morphology indicating a loss of their fibroblast characteristic morphology that is pronounced in astrocytic monocultures and differs widely from *in vivo* astrocytes [35]. These changes are also visible on the proteomics level. We find Fibronectin expression drastically reduced in cocultured astrocytes and observe a reduced expression of the astrocyte-specific intermediate filament Glial fibrillary acidic protein (GFAP). This supports the apparent changes in morphology and underlines the obvious differences of astrocytic cells obtained by both culture methods (Fig 1A and 1B).

To analyze whether astrocytes react to changes in neuronal signaling we stimulated neuron-glia cocultures with BDNF, a neurotrophic factor that has been shown to induce local protein synthesis in neuronal dendrites [36]. We monitored ongoing protein translation dynamics by coapplication of AHA and subsequent 'click reaction' covalently linking a Tetramethylrhodamine (TAMRA)-alkyne tag to the integral azide group of AHA [22].

**Fig 2:**
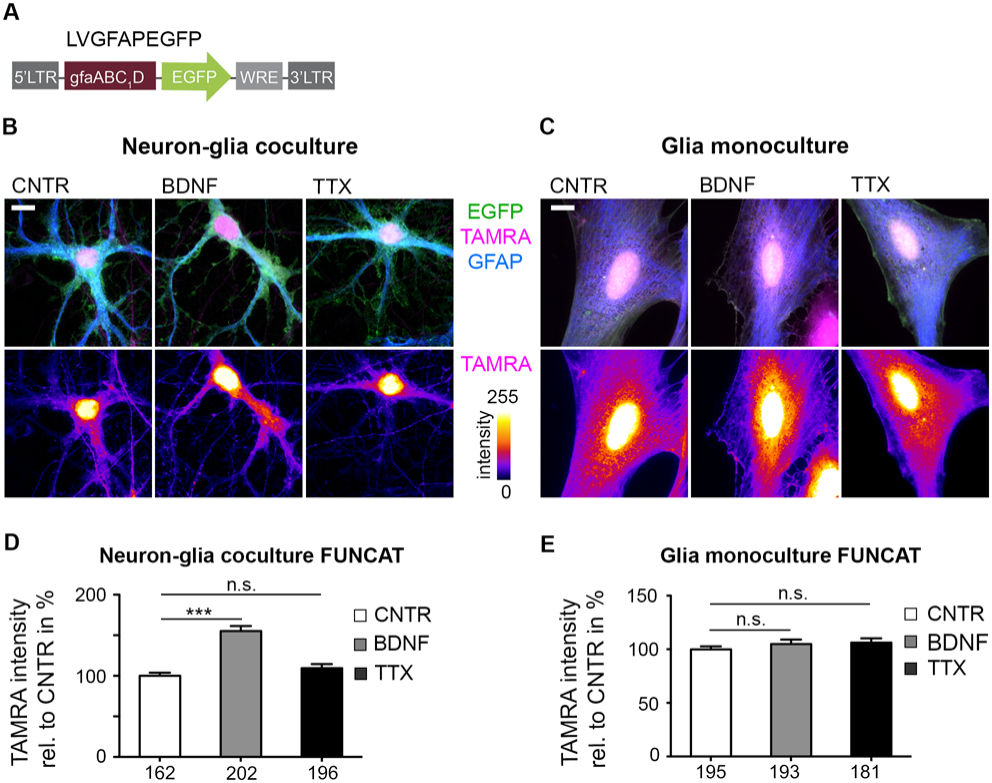
Up-regulation of BDNF-induced astroglial protein synthesis depends on the presence of neurons. (A) Schematic drawing of the lentiviral expression vector LVGFAPEGFP that expresses EGFP cell type-specifically in astrocytes under control of the simplified GFAP promoter version gfaABC_1_D. (B) Astrocytes expressed EGFP cell type-specifically after infection with LVGFAPEGFP in a neuron-glia coculture (DIV 22). Cultures were treated for 4 h with either 50 ng/ml BDNF or 2 μM TTX in the presence of 4 mM AHA and processed for 'click reaction' with a TAMRA-alkyne tag in the FUNCAT procedure. BDNF application led to an upregulation of TAMRA signal intensities up to 150%. TAMRA intensities are color coded in the lower panel (scale bar = 10 μm). (C) GFAP-positive astrocytes in monoculture (DIV 20–22) expressing EGFP show fibroblast-like morphology. No differences in TAMRA signal intensities were observed when cultures were treated analogous to (B) (scale bar = 10 μm). (D) Cultures were manipulated as described in (B). Mean TAMRA signal intensities were determined within the EGFP mask. A significant increase in TAMRA signal intensities was detected after BDNF treatment. Numbers at the X-axis indicate the number of cells included in the quantification (3 independent experiments; represented data are mean +/- SEM; ONE-way ANOVA, ***: p<0.0001). (E) TAMRA signal intensities were quantified in GFAP-positive astrocytes expressing EGFP according to (D). No significant differences in signal intensities after either BDNF or TTX treatment were observed (3 independent experiments; represented data are mean +/- SEM; ONE-way ANOVA, p>0.05).

Indeed, we identified a fraction of GFAP positive astrocytes that show an increase of TAMRA-tagged, newly synthesized proteins with high TAMRA signals in or close to the nucleus spreading also into astrocytic processes (Fig 2B). In order to quantify the obtained differences in TAMRA signal intensities, we infected neuron-glia cocultures with the lentivirus LVGFAPEGFP. This virus mediates a cell type-specific expression of EGFP in astrocytes utilizing a basic GFAP promoter version (Fig 2A) [29,30]. The EGFP expression was used as a mask to determine the outline of the astrocytic cell for TAMRA signal intensity evaluation. We found *de novo* synthesized proteins upregulated to 150% of control levels (CNTR), whereas a treatment with tetrodotoxin (TTX), used to block neuronal activity, did not lead to significant changes (Fig 2B and 2D). In parallel, we performed an analogous set of experiments with astrocytes kept in monocultures. Coapplication of BDNF or TTX with 4 mM AHA and subsequent visualization with a TAMRA-alkyne tag did not reveal any differences in translation rates arguing for a directly or indirectly induced effect of BDNF that is mediated by neurons (Fig 2C and 2E).

### Cell type-specific labeling of newly synthesized proteins with ANL and mMetRS^L274G^

Metabolic labeling of *de novo* synthesized proteins using non-canonical amino acids such as azidohomoalanine (AHA) is an effective method to analyze dynamic cellular proteomes, but it does not allow for any distinction of cell type-specific proteomes in a complex cellular environment [32,37,38].

**Fig 3:**
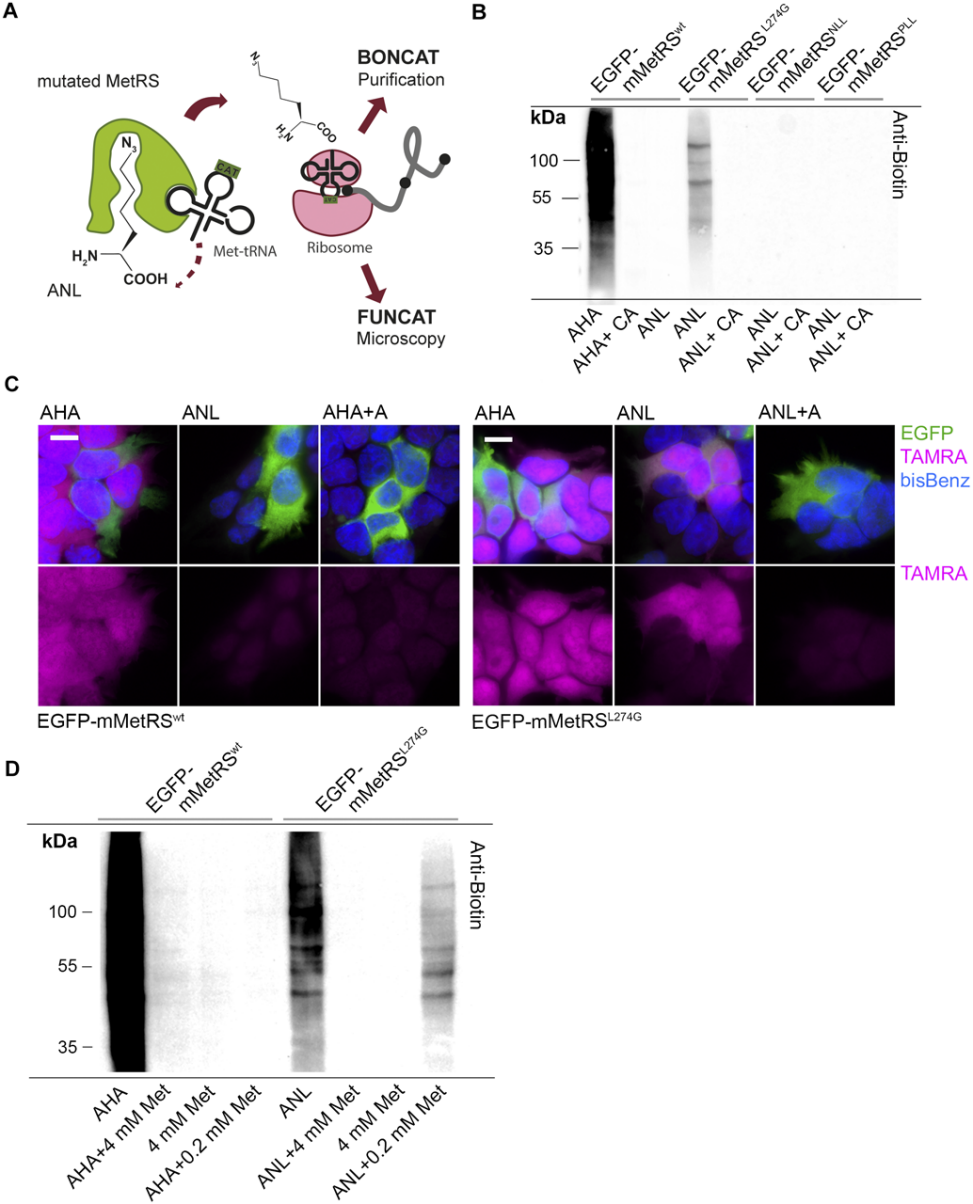
Cell type-specific protein labeling with ANL and a mutated *Mus musculus* MetRS. (A) ANL carries an enlarged side chain and therefore cannot fit into the methionine binding pocket of the wild type MetRS. An enlargement of the methionine binding pocket by a single amino acid exchange allows ANL binding and coupling to the Met-tRNA. (B) HEK293T cells overexpressing EGFP-tagged wild type mMetRS^wt^ or the mutated versions mMetRS^NLL^, mMetRS^PLL^ and mMetRS^L274G^ were subjected to either 4 mM ANL, AHA or Met in Met-free medium for 1 h. Successful ANL integration into proteins was detected with a biotin-alkyne tag by performing a ‘click-reaction’ of cell lysates, followed by immunodetection of biotinylated proteins. Whereas the endogenous MetRS can process AHA, ANL integration is specific for cells carrying the mutated version EGFP-mMetRS^L274G^ only. Blockade of protein synthesis with 100 μg/ml cycloheximide and 40 μM anisomycin (CA) prevents ANL integration and subsequent biotin tagging. Very weak to none-tagged biotin was observed for both EGFP-mMetRS^NLL^ and EGFP-mMetRS^PLL^ expressing cells. (C) HEK293T cells overexpressing either EGFP-mMetRS^wt^ or EGFP-mMetRS^L274G^ were incubated for 1 h with either 4 mM AHA or ANL in Met-free medium with or without 40 μM anisomycin (A). Cells were fixed and AHA or ANL labeled proteins were visualized with a TAMRA-alkyne tag in the ‘click reaction’. TAMRA-positive signals are specific for cells expressing EGFP-mMetRS^L274G^ with comparable efficiency to AHA incorporation by the wild type MetRS. Notably, TAMRA signals are detectable within the entire cell. Moreover, metabolic incorporation of ANL can be blocked by anisomycin (A). Nuclei are stained with bis-Benzimide (bisBenz) (scale bar = 10 μm). (D) HEK293T cells overexpressing EGFP-mMetRS^wt^ or EGFP-mMetRS^L274G^ were incubated for 1 h with 4 mM Met, AHA or ANL either without Met or in combination with 4 mM Met in Met-free medium or standard cell culture medium containing 0.2 mM Met. Successful AHA or ANL integration was visualized by tagging with a biotin-alkyne tag. Low or equimolar Met concentrations reduced biotin-tagged proteins after AHA inoculation, whereas a biotin signal is observable after ANL integration. Although with reduced efficiency, ANL labeling is still possible under conditions where low concentrations of Met are present.

To analyze proteome changes exclusively in astrocytes we developed a metabolic labeling technique that is able to track *de novo* synthesized proteins in a cell type-specific fashion and that allows further purification, isolation and identification of labeled proteins. This technique is based on previously described fluorescent or bioorthogonal non-canonical amino acid tagging methods (BONCAT and FUNCAT) utilizing non-canonical amino acids and the cells own protein synthesis machinery to integrate amino acids analogues into proteins [16,22]. To restrict metabolic labeling to single cell types we made use of the long-chained amino acid azidonorleucine (ANL), which can only be processed by a methionyl-tRNA synthetase (MetRS) that possesses an enlarged methionine-binding pocket able to bind ANL [23,24]. This enzyme, therefore, allows cell type-specific ANL incorporation using cell type-specific promoters such as the GFAP promoter (Fig 3A; S1 Fig). In order to enlarge the Met binding pocket of the *Mus musculus* MetRS (mMetRS) we modified amino acid residues analogous to *Escherichia coli* MetRS mutants that were found to be highly effective in binding ANL (S1 Fig) [23,24,39]. EGFP-fusion proteins of the three murine MetRS versions EGFP-mMetRS^L274G^, -mMetRS^NLL^, and -mMetRS^PLL^, comprising a single or different combinations of amino acid exchanges within the Met-binding pocket, were expressed in HEK293T cell cultures and tested for their capability to bind and activate ANL. Cells were incubated with ANL in medium deprived of Met and the successfully integrated ANL residues were subsequently covalently coupled to a biotin-alkyne tag via 'click reaction' followed by an immunoblotting analysis of tagged cell lysates.

A prominent biotin signal encompassing different molecular weight proteins was achieved when cells overexpressed EGFP-mMetRS^L274G^ (Fig 3B). Either inhibition of protein synthesis or overexpression of mMetRS^wt^ strongly reduced or prevented ANL labeling. In comparison to mMetRS^L274G^ the results for both MetRS versions mMetRS^NLL^ and mMetRS^PLL^ were less promising: Protein lysates revealed little to none biotin-positive signals demonstrating that these MetRS mutants were inefficient in activating ANL in this system (Fig 3B). In order to validate that only cells carrying the EGFP-mMetRS^L274G^ would integrate ANL, we used a TAMRA-alkyne tag in the 'click reaction' to observe ANL integration on the single cell level by fluorescence microscopy. Indeed, only HEK293T cells overexpressing the mutated enzyme revealed positive TAMRA signals both in the cytoplasm and in nuclei of the cells whereas overexpression of the MetRS wildtype version gained no TAMRA-tagged proteins (Fig 3C).

To apply this technology *in vivo*, ANL activation and subsequent integration into proteins must tolerate the presence of methionine. In order to test this scenario in cell culture, we overexpressed EGFP-mMetRS^L274G^ in HEK293T cells and administered 4 mM ANL for 1 h in the presence of 0 mM, 0.2 mM, or 4 mM Met. Whereas AHA integration is completely blocked by low concentrations of Met, labeling with ANL occurred in the presence of Met albeit with a reduced efficiency, suggesting that mMetRS^L274G^ has a lower affinity for Met after enlargement of the substrate binding pocket than the wild type MetRS version (Fig 3D).

To ensure that ANL-labeled proteins reflect the pool of proteins synthesized in cells under normal conditions, we further characterized cell viability and stability of proteins that exhibited ANL incorporation. Although we observed a trend for a slight increase in cell death, the number of dead HEK293T cells within the culture is low despite the high metabolic rate in this cell type (S2 Fig). Furthermore, to exclude an increase of misfolded proteins by ANL integration, we analyzed the pool of ubiquitinated proteins by immunoprecipitation with an antibody against Ubiquitin in HEK293T cells that integrated ANL. We found similar levels of ubiquitinated proteins comparable to control conditions indicating correct folding of proteins that integrated ANL (S2 Fig).

**Fig 4:**
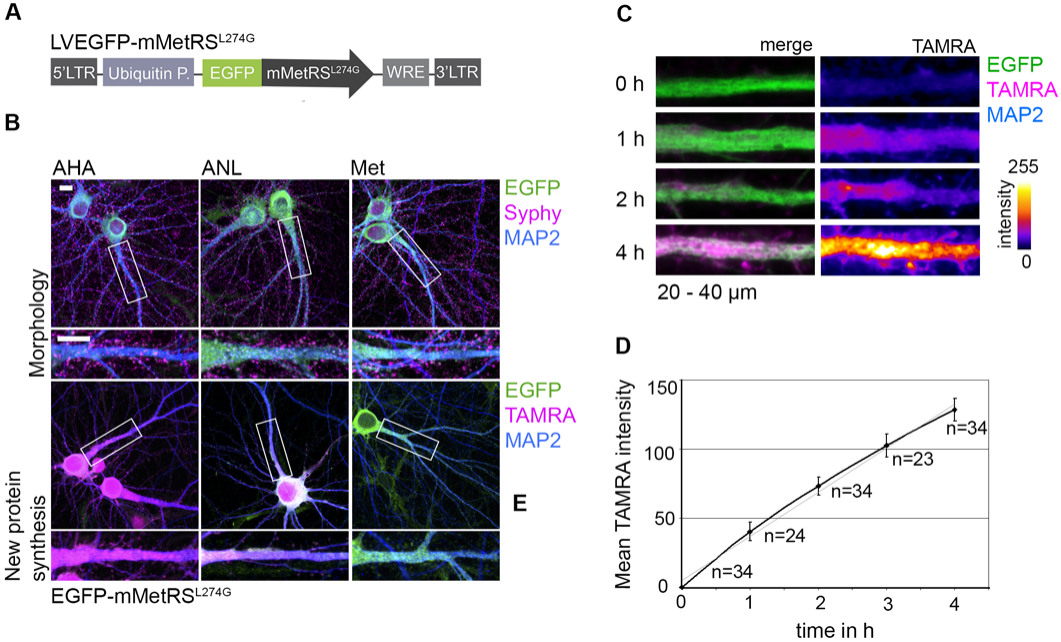
ANL labeling in neurons. (A) Scheme of the lentiviral construct driving the non-cell-selective expression of EGFP-mMetRS^L274G^ under the ubiquitin promoter (LVEGFP-mMetRS^L274G^). (B) Primary neuron-glia cocultures (DIV 22), infected with LVEGFP-MetRS^L274G^ were labeled for 4 h with either 4 mM ANL, AHA or Met. Subsequent immunostaining of MAP2 and Synaptophysin (Syphy) or visualization of ANL- or AHA-labeled proteins by ‘click reaction’ revealed an intact morphology of ANL-labeled cells indistinguishable from AHA or Met treated controls. TAMRA-positive signals are evident in neuronal dendrites and spines. Segments of dendrites in the detail were straightened and magnified (scale bar = 10 μm). (C) Neurons of a cortical neuron-glia coculture (DIV 22) that express EGFP-mMetRS^L274G^ were labeled with 4 mM ANL for incubation times ranging from 0 h to 4 h. Newly synthesized proteins that integrated ANL were visualized with the TAMRA-alkyne tag by ‘click reaction’ and subsequent immunocytochemistry. Examples of representative 20 μm dendritic sections after the 'click reaction' display constant increases of TAMRA intensity in neuronal dendrites with ANL labeling duration ranging from 0 to 4 h. TAMRA stainings are intensity coded in the right panel. (D) Mean fluorescence intensities of ANL-labeled proteins were measured within the main dendrite in a section ranging from 20 μm–40 μm of the proximal end of the dendrite using the MAP2 staining as a mask. Mean fluorescence intensities in dendrites increased constantly over the observed time range suggesting a continuous integration of ANL into newly synthesized proteins over time. Numbers represent the number of analyzed dendrites (represented data are mean +/- SEM of 3 independent experiments).

To test the applicability of this cell type-specific labeling technique in the murine neural cell culture model, we utilized neuron-glia cocultures from rats and expressed EGFP tagged mMetRS^L274G^ ubiquitously in both neurons and astrocytes by a lentiviral expression system (Fig 4A) [29]. Infected cultures were incubated with ANL, AHA or Met for 4 h and newly synthesized proteins were subsequently visualized using a TAMRA-alkyne tag. The majority of ANL-labeled proteins were detected in neuronal dendrites and spines (Fig 4B). Neither the integrity of neuronal dendrites, revealed by an immunostaining against the neuronal intermediate filament Microtubule-associated protein 2 (MAP2), nor the abundance of presynaptic terminals, marked by anti-Synaptophysin (Syphy) immunostaining, exhibited any alterations when compared to Met treated controls, indicating that also in this model system metabolic labeling with ANL is conceivable.

In order to investigate whether cellular processes are affected by ANL incorporation or Met depletion, we analyzed the dynamics of protein translation representing one of the major biosynthetic cellular processes. For that, we infected neuron-glia cocultures with LVEGFP-mMetRS^L274G^, incubated the cells with ANL over a time range from 0 to 4 h and monitored the increase of labeled proteins in neurons using a TAMRA-alkyne tag in a subsequent ‘click-reaction’. Constant increases of TAMRA signals were observed in dendritic segments demonstrating continuous integration of ANL into proteins over time indicative for an intact protein synthesis process (Fig 4C and 4D).

**Fig 5:**
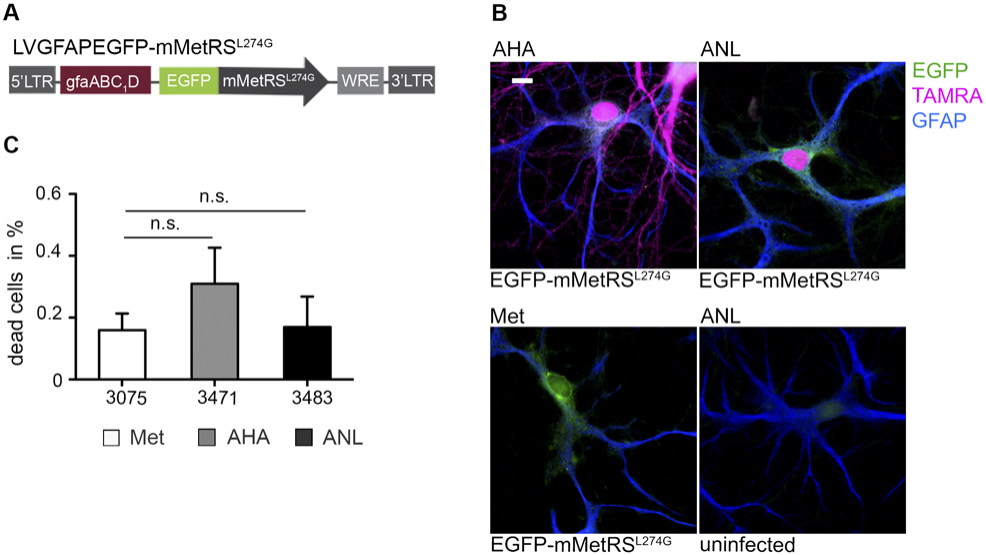
ANL labeling in astrocytes. (A) Scheme of the lentivirus construct LVGFAPEGFP-mMetRS^L274G^ driving the expression of EGFP-mMetRS^L274G^ from the simplified GFAP promoter version gfaABC_1_D. (B) Primary neuron-glia cocultures (DIV 14), infected with the lentivirus LVGFAPEGFP-mMetRS^L274G^, expressed the enzyme cell type-specifically in astrocytes. Cultures were incubated with either 4 mM AHA, ANL or Met for 4 h in Met-free medium, and AHA or ANL incorporation was visualized using a TAMRA-alkyne tag in a subsequent ‘click-reaction’. Astrocytes are identified employing GFAP immunostaining. AHA labeling occurred in a non-cell-selective manner both in neurons and astrocytes. TAMRA-positive proteins are detected solely in GFAP-positive, EGFP-mMetRS^L274G^ expressing astrocytes upon incubation with ANL. Comparable TAMRA signals are observable in ANL and AHA labeled cells with a high nuclear and cytoplasmatic intensity of tagged proteins (scale bar = 10 μm). (C) Primary neuron-glia cocultures were infected with LVGFAPEGFP-mMetRS^L274G^ to express EGFP-mMetRS^L274G^ in astrocytes. Cocultures (DIV 22) were subjected to either 4 mM AHA, ANL or Met in Met-free medium for 4 h. Live-staining with 2 μg/ml propidium iodide for dead or dying cells revealed a non-significant increase in the percentage of dead cells of the counted EGFP-mMetRS^L274G^-positive astrocyte population after ANL incubation. Numbers at the X-axis indicate the total number of EGFP-mMetRS^L274G^ positive astrocytes counted (n = 5 independent experiments; represented data are mean +/- SEM; ONE-way ANOVA, p>0.05).

For a transfer of the cell type-specific labeling technique to astrocytes within a neuron-glia coculture we generated the lentivirus LVGFAPEGFP-mMetRS^L274G^ by replacement of the ubiquitin promoter with the reduced GFAP promoter version gfaABC_1_D, which was recently shown to be a specific promoter for the expression of proteins in astrocytes (Fig 5A) [30]. Also here, infected neuron-glia cocultures were incubated with ANL and *de novo* synthesized proteins were detected with a TAMRA-alkyne tag. We observed TAMRA signals in the soma and the nucleus of single astrocytes expressing EGFP-mMetRS^L274G^. Application of ANL to cells not expressing mMetRS^L274G^, being either uninfected astrocytes or neurons in immediate contact to infected astrocytes, did not result in TAMRA-tagged proteins in these cells, demonstrating the high specificity of the labeling method (Fig 5B and S5 Fig). Similar results were obtained when a biotin-alkyne tag was used in the 'click chemistry' reaction (S3 Fig). Again, we investigated the influence of ANL labeling on astrocyte viability by a live propidium iodide staining to visualize dead or dying cells. For that, we overexpressed EGFP-mMetRS^L274G^ in astrocytes of a neuron-glia coculture and applied ANL for 4 h. We observed a slight but non-significant increase in dead cells compared to the Met control indicating a low stress level of cells after ANL integration (Fig 5C).

### Astrocytes reveal changes in protein expression upon BDNF application

Next we applied the cell type-specific metabolic labeling technique to analyze the molecular consequences of BDNF application on astrocytes more closely, thereby disregarding neuronal protein synthesis. For this, we tested candidate proteins for possible increases in protein synthesis rates after BDNF application. As a proof of principle, we chose the gap junction subunit Connexin-43 (Cx43) that is expressed in astrocytes but not in neurons [40]. Thus, an altered expression analysis can be done in principle with conventional methods. In contrast to Cx43, we opted for the 60S ribosomal protein L10a (Rpl10a) as our second candidate. Rpl10a is a ribosomal subunit ubiquitously expressed in both neurons and astrocytes. Hence, possible changes in expression levels cannot be readily assigned to either neurons or astrocytes, and, therefore, are in need for a method that allows cellular distinction.

**Fig 6:**
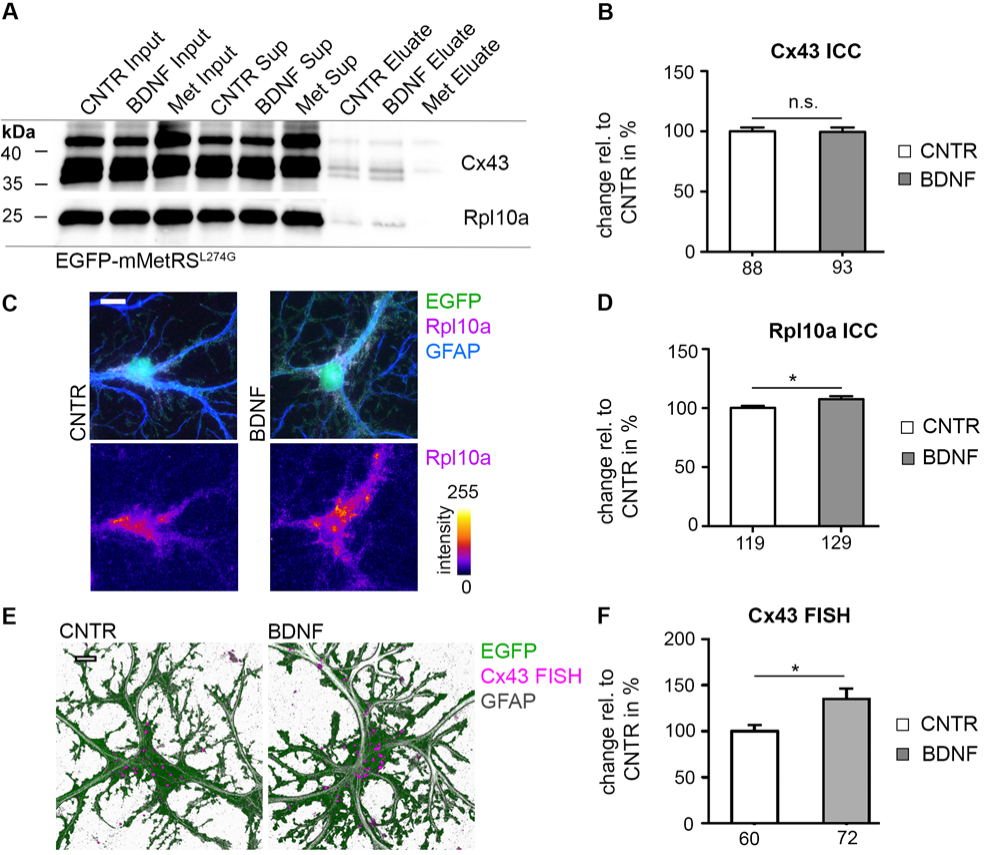
Upregulation of candidate proteins upon BDNF treatment in astrocytes. (A) Neuron-glia cocultures (DIV 22), infected with LVGFAPEGFP-mMetRS^L274G^, were incubated with 4 mM ANL or 4 mM Met (Met) for 4 h with (BDNF) or without 50 ng/ml BDNF (CNTR). Cell lysates were tagged with a biotin-alkyne tag via 'click-chemistry'. Biotin-tagged proteins were affinity purified with NeutrAvidin agarose. ANL incorporation and subsequent biotin tagging allows enrichment of *de novo* synthesized proteins compared to the Met incorporation control (Met Eluate). Overall protein levels of Cx43 and Rpl10a are comparable among the three different conditions in the lysates (Input) representing the joined pool of both pre-existing and *de novo* synthesized proteins as well as in the unbound fraction (Sup). In contrast, BDNF application resulted in increased levels of *de novo* synthesized and thus biotin-tagged Cx43 (43 kDa; both lower bands represent unphosphorylated and monophosphorylated Cx43) and Rpl10a (25 kDa) on immunoblots after purification (Eluate; 3 independent experiments). (B) Neuron-glia cocultures (DIV 22) were treated with 50 ng/ml BDNF for 4 h. A positive immunocytochemical GM130 staining for the Golgi apparatus (ICC) was used as a mask to quantify Cx43 signal intensity. No difference in Cx43 signal intensities was found. Numbers at the X-axis indicate the number of cells included in the quantification (3 independent experiments, represented data are mean +/- SEM, student's t-test, p>0.05). (C) Neuron-glia cocultures (DIV 22) were infected with LVGFAPEGFP and treated with 50 ng/ml BDNF for 4 h. Elevated Rpl10a positive signals were observed in GFAP-positive astrocytes by applying immunocytochemistry for Rpl10a and GFAP. Rpl10a signal intensities are color coded in the lower panel (scale bar = 10 μm). (D) Quantification of Rpl10a signal intensities, obtained as in (C), was done within the EGFP mask. A significant increase in Rpl10a signal intensity was observed. Numbers at the X-axis indicate the number of cells included in the quantification (5 independent experiments, represented data are mean +/- SEM, student's t-test, *: p<0.05). (E) Neuron-glia cocultures were infected with LVGFAPEGFP and treated with 50 ng/ml BDNF for 1 h. FISH procedure was conducted on fixed samples and revealed an increase of Cx43 mRNA-positive punctae in BDNF treated cells (scale bar = 10 μm). Images are depicted as 3D-reconstructions. FISH positive punctae were enlarged for representative purposes. (F) Quantification of Cx43 mRNA-positive punctae, obtained as described in (E), exhibited a significant increase in BDNF treated cultures. Numbers at the X-axis indicate the number of cells included in the quantification (3 independent experiments, represented data are mean +/- SEM, student's t-test, *: p<0.05).

In order to investigate consequences of BDNF treatment on the astrocytic proteome, neuron-glia cocultures, infected with LVGFAPEGFP-mMetRS^L274G^, were treated with BDNF for 4 h in combination with ANL labeling. After cell lysis, lysates were tagged to a biotin-alkyne tag using 'click reaction'. In order to identify changes in protein translation rates of Cx43 and Rpl10a, biotin-tagged d*e novo* synthesized proteins were subsequently isolated from untagged proteins by NeutraAvidin agarose affinity purification and analyzed by immunoblotting. Whereas the overall pool of Cx43 and Rpl10a in cell lysates (Input; Fig 6A and S6 Fig) reveal no change of protein amounts between untreated and BDNF treated groups, both *de novo* synthesized astrocytic Cx43 and Rpl10a were found to be upregulated after BDNF treatment in the isolated pool of *de novo* synthesized and, thus, biotin-tagged proteins (Eluate).

In order to confirm the previous results we analyzed possible changes in overall protein amounts using immunocytochemistry. Astrocytes of neuron-glia cocultures were filled with EGFP by infection with LVGFAPEGFP, and Rpl10a signal intensities were analyzed within the EGFP mask. Rpl10a signal intensity significantly increased after treatment with BDNF (Fig 6C and 6D). Similar experiments conducted for *de novo* synthesized Cx43 within the Golgi apparatus did not reveal differences in Cx43 signal intensities after BDNF application (Fig 6B and S4 Fig). Because a distinction between *de novo* synthesized, thus ANL labeled proteins, and any pre-existing proteins is not possible by this conventional kind of immunocytochemistry, BDNF-derived effects could possibly not be resolved by this methodical approach. Therefore, we analyzed Cx43 expression at the mRNA level. After BDNF treatment for 1 h we applied fluorescence *in situ* hybridization (FISH) and observed an up-regulation in the number of Cx43 mRNA-positive punctae in astrocytes of a neuron-glia coculture indicating a BDNF dependent up-regulation of Cx43 mRNA (Fig 6E and 6F).

These first experiments prove that this technique allows the analysis of protein dynamics in a cell type-distinctive fashion, and therefore, provides a basis for in depth analysis with a higher resolution than conventional proteomic techniques can provide.

## Discussion

### Cell type-selective tagging of *de novo* synthesized proteins using ANL and the *Mus musculus* MetRS^L274G^

Astrocytes and neurons are thought to tightly cooperate to support not only the development but also the diverse functions of either cell type after maturation of neural circuits, thereby, being efficient members of a high-performance cellular network. Whereas the neuronal part has been and still is minutely investigated, the role of astrocytes in this duet is rather poorly understood. This is partly due to the lack of appropriate methods enabling cell type-specific differentiation of astrocytes and neurons while keeping their physical and physiological communication and association intact.

Metabolic labeling with bioorthogonal amino acids is a versatile method to characterize proteomes owing to the minor cellular manipulations required, the tight temporal control of labeling timing and the functionality supplied by integral azide or alkyne groups. These functionalities allow detection by different means including visualization and isolation with conventional cell biological techniques [26,37,41]. Nevertheless, this method is not suitable to disentangle heterogeneous protein pools of complex organs or multicellular organisms with cell type-specific resolution as, for instance in the brain, astrocytes and neurons share a large pool of identical proteins. In order to confine metabolic labeling to specific cell types we opted to use the non-canonical amino acid ANL in combination with cell restricted expression of a mutated MetRS, which is able to couple ANL to the respective tRNA^Met^ [23–27].

In previous studies, a mutant of the *Escherichia coli* methionyl-tRNA synthetase, the NLL-EcMetRS, was generated that binds ANL and allows the replacement of an initiator-methionine by ANL during protein synthesis [24,25]. To circumvent the limitation introduced by initiator-methionine labeling, we looked for mutations within the binding pocket of the *Mus musculus* MetRS (mMetRS), which allow also for internal ANL labeling [23,24]. Three modified forms that were efficient to bind ANL in the *Escherichia coli* system were tested but only the mMetRS^L274G^ resulted in significant protein labeling (Fig 3B and 3C).

Interestingly, the mouse version of the potent bacterial ANL activator NLL-EcMetRS was not able to activate ANL in sufficient amounts demonstrating that, although the sequence of the binding pocket is strongly conserved among species, differences in ANL activation are found among MetRS mutants of different species (Fig 3B and S1 Fig). This is consistent with results from *Drosophila melanogaster*, where an analogous MetRS mutant, namely MetRS^L262G^, is less efficient in activating ANL than the mouse MetRS^L274G^ [26]. Importantly, mMetRS^L274G^ shows an apparent lower affinity for Met than the corresponding wild type enzyme (mMetRS^wt^) offering the possibility to conduct metabolic labeling of proteins with ANL in the presence of physiological Met concentrations in cell culture, a promising feature for future *in vivo* applications (Fig 3D).

Notably, we transferred this labeling technique to a neuron-glia coculture model system. By restricting expression of MetRS^L274G^ to astrocytes we labeled *de novo* synthesized proteins in these cells both for visualization purposes as well as for purification and subsequent detection by immunoblotting. We thereby proof that cell-selective metabolic labeling utilizing ANL in combination with MetRS^L274G^ is also feasible for complex multi-cell type culture systems and might be applicable for any heterogeneous cellular system once a convenient cell-selective promoter is at hand (Fig 5 and S3 Fig).

Furthermore, we tested for global toxicity induced by ANL application both in HEK293T cells and in neuron-glia cocultures and observed no significant effects on cell survival, cell viability, or protein degradation and translation events (Figs 4, 5 and S2 Fig). These observations are supported by a recent study employing chronic ANL feeding, which resulted in viable *Drosophila melanogaster* flies with limited side effects concerning survival rates and behavior assays [26].

Altogether, the benefits of this technique, including high specificity, cell selectivity, diversity of applicable tags, and temporal control, make this a versatile method for the exploration of astrocytic proteome dynamics in complex neuronal networks and also for other questions that are based on a cooperation between different cell types and that require cellular distinction.

### Applying cell type-selective labeling with ANL within the context of neuron-glia interaction and neuronal signaling

Neurons and astrocytes cooperate to fine-tune neuronal activity and to enable mechanisms including synaptic plasticity [42,43]. One important mechanism by which short-term changes in synaptic strength are consolidated to long-term changes is the dynamic modulation of the neuronal proteome via protein translation. In contrast to an emerging literature on neuronal proteome dynamics, there is a paucity of information regarding proteome dynamics in astrocytes. Thus far, efforts to examine astrocytic proteomes have involved a physical separation of astrocytes from neurons or genetic manipulation resulting in an analysis most likely reflecting a non-physiological context [44,45]. In order to analyze astrocyte-specific proteome dynamics within a heterogeneous cellular system we utilized cell type-restricted mMetRS^L274G^ expression and ANL labeling. This technique now allows us to easily define the time frame for protein labeling and to isolate tagged proteins in our model system. These are benefits that enable this technique to augment current available tools addressing the issue of astrocytic proteome dynamics.

We used mMetRS^L274G^ cell type-specific expression in astrocytes in conjunction with ANL to identify astroglial Connexin-43 (Cx43) and 60S ribosomal protein L10a (Rpl10a) expression upregulated upon BDNF treatment in neuron-glia cocultures (Fig 6A–6F). Although the analysis of Cx43 expression did not require cell type-specific labeling, it provides a proof of principle for the analysis of astroglial *de novo* protein synthesis. It is important to note that the increased pool of newly synthesized Cx43 can be easily masked when the absolute Cx43 pool within the Golgi apparatus is analyzed with conventional immunocytochemistry (see Fig 6B and S5 Fig). In line with our findings, Cx43 expression has been described previously to be influenced by neurons [46,47]. A regulatory impact on Cx43 expression by neurons is consistent with the suggested role of astrocytes in stabilizing neuronal network activity, since Cx43 has been shown to modulate glutamatergic synaptic activity of hippocampal CA1 pyramidal cells by an as yet unknown mechanism [48].

In addition, we identified an up-regulation of astrocytic Rpl10a expression in response to BDNF presentation with our cell type-selective tagging technique and validated this finding by immunocytochemistry (Fig 6C and 6D). The up-regulation of both candidate proteins is small but significant and these effects would most likely be undetectable if cell type-specific labeling was not used. In addition, since BDNF actions at the neuronal site are manifold, the astrocytic origin of both analyzed proteins is essential to prove, but is difficult to assess with conventional methods [49]. Using the specific expression of mMetRS^L274G^ in GFAP-positive astrocytes we can directly reason that the labeled proteins are of astrocytic origin.

So far we cannot exclude that the observed effects are directly regulated by BDNF, since astrocytes express the truncated version of the TrkB-receptor TrkB-T1 that has been shown to execute signaling activity in cultured astrocytes and in astrocytes of adult rat brain slices [50,51]. The absence of BDNF induced effects in astrocytic monocultures strongly points towards an influential role of neurons either by a change of the astrocytic phenotype or by a BDNF-driven activation of neuronal signaling cascades that feedback to astrocytes. Whereas previously reported changes of astrocytic GFAP and S100-B levels in a rat model of depression relinquished a cell type-specific analysis of involved signaling pathways, studies in the retinal glia cell prove the active role of glial TrkB receptors in BDNF signaling by a cell-selective knockout model [52,53]. Therefore, cell type-specific analysis tools are a prerequisite to elucidate the role of BDNF signaling on astrocytic function in the nervous system.

## Supporting Information

S1 Fig The methionine binding pocket of MetRS is highly conserved among species. (A) Whereas AHA is sterically similar to methionine and can be processes by the endogenous methionyl-tRNA synthetase, the side chain of ANL is longer and, therefore, excluded by the wild type enzyme. (B) The Met binding pocket is highly conserved and sequences are comparable between the *Escherichia coli* and *Mus musculus*. Amino acids that substantially shape the binding pocket are highlighted in pink. To allow for binding of the long-chained ANL, three *Mus musculus* MetRS (mMetRS) mutants were generated, one being created by a single amino acid exchange at position 274 leading to mMetRS^L274G^ (highlighted in green). Two more combinations of amino acid exchanges led to mMetRS^PLL^ and mMetRS^NLL^. (C) Labeling of *de novo* synthesized proteins can be done in a non-cell-selective fashion with bioorthogonal, non-canonical amino acids such as the azide-bearing azidohomoalanine (AHA). AHA is utilized by the endogenous methionyl-tRNA synthetase (MetRS) and replaces methionine (Met) during the course of protein translation. Due to the azide-harboring side chain of AHA, AHA-carrying proteins can be visualized by covalent reaction to a fluorescent-alkyne tag (FUNCAT) or purified via a biotin-alkyne tag (BONCAT) using ‘click reaction'. Processing of azidonorleucine (ANL) affords a mutated form of the MetRS with an enlarged Met binding pocket that allows ANL to be coupled to the respective tRNA^Met^. When cells are genetically manipulated to express the mutated MetRS, cell type-specific ANL processing and integration of ANL into newly synthesized proteins becomes feasible. ANL labeling in astrocytes can be achieved with expression of the mutated MetRS under a promoter for specific astrocytic proteins like GFAP, respectively. Also here, the terminal azide group within ANL allows visualization or purification of labeled proteins following 'click reaction'.
(TIF)

S2 Fig Analysis of cell death and protein degradation proves a vital culture after ANL integration. (A) HEK293T cells were transfected with either the EGFP-mMetRS^wt^ or the mutant enzyme version EGFP-mMetRS^L274G^ and incubated for 4 h with 4 mM ANL or Met in Met-free medium. A live-staining with a 2 μg/ml propidium iodide solution revealed a slight tendency towards an increase of dead cells with propidium iodide positive nuclei in cells expressing EGFP-mMetRS^L274G^ and ANL treatment compared to Met or EGFP-mMetRS^wt^ control in HEK293T cells. The percentage of dead cells refers to the total number of counted cells as indicated at the X-axis (represented data are mean +/- SEM; n = 5 independent experiments; One-way Anova, p>0.05). (B) HEK293T cells overexpressing EGFP or EGFP-mMetRS^L274G^ were incubated with either 4 mM Met or ANL in Met-free medium for 2 h. Cell lysates underwent immunoprecipitation applying an anti-Ubiquitin antibody to pull down ubiquitinated proteins. No changes of ubiquitinated protein levels in cells that overexpressed EGFP-mMetRS^L274G^ and that are incubated with ANL are revealed when compared to Met-treated controls. Both images originate from the same blot.
(TIF)

S3 Fig Successful ANL integration into *de novo* synthesized proteins by BONCAT. Primary neuron-glia cocultures (DIV 21), either infected with LVGFAPEGFP-mMetRS^L274G^ or not treated, were incubated with either 4 mM AHA, ANL or Met for 4 h. Successful ANL integration into proteins was detected with a biotin-alkyne tag using ‘click-reaction’ of cell lysates, followed by immunodetection of biotinylated proteins. Also here, ANL integration is specific for cultures expressing EGFP-mMetRS^L274G^ only.
(TIF)

S4 Fig Example of Cx43 quantification in the Golgi apparatus of astrocytes. Neuron-glia cocultures (DIV 22) were infected with LVGFAPEGFP and treated with 50 ng/ml BDNF for 4 h. Cx43 positive signals were observed in the Golgi apparatus of astrocytes applying immunocytochemistry for Cx43 and the Golgi marker GM130. Cx43 signal intensities are color coded in the lower panel. No increase of *de novo* synthesized Cx43 within the Golgi apparatus after BDNF application was observed (scale bar = 10 μm).
(TIF)

S5 Fig Cell type-specific labeling with ANL in a neuron-glia coculture. Primary neuron-glia cocultures (DIV 22), infected with the lentivirus LVGFAPEGFP-mMetRS^L274G^, expressed the enzyme cell type-specific in astrocytes. Cells were incubated with either 4 mM AHA, ANL or Met for 4 h in Met-free medium and AHA or ANL incorporation was visualized by a TAMRA-alkyne tag. AHA labeling occurred in a non-cell-selective manner both in neurons and astrocytes. TAMRA-positive proteins are detected solely in EGFP-mMetRS^L274G^ expressing astrocytes when labeled with ANL. MAP2 positive neurons integrate AHA into *de novo* synthesized proteins whereas only background staining is found in neurons incubated with ANL or Met (scale bar = 10 μm)
(TIF)

S6 Fig Original images of Immunoblotting. (A) Original immunoblots related to Fig 1B. (B) Original immunoblots related to Fig 3B. The layer with the protein ladder was omitted in the main figure. (C) Original immunoblots related to Fig 3D. The layer with the protein ladder was omitted in the main figure. (D) Original immunoblots related to Fig 6A. The layer with the protein ladder was omitted in the main figure. The lower image represents the same blot with lower exposure to display both input and supernatant fractions that are enhanced in the main figure. (E) Original immunoblots related to S2 Fig. (F) Original immunoblots related to S3 Fig.
(TIF)

S1 File Supplemental Material and Methods. (DOC)
