# Supplementary figures and images for: Monitoring Astrocytic Proteome Dynamics by Cell Type-Specific Protein Labeling
Source: PLoS One. 2015 Dec 21;10(12):e0145451. doi: 10.1371/journal.pone.0145451 (PMC4686566; doi:10.1371/journal.pone.0145451)

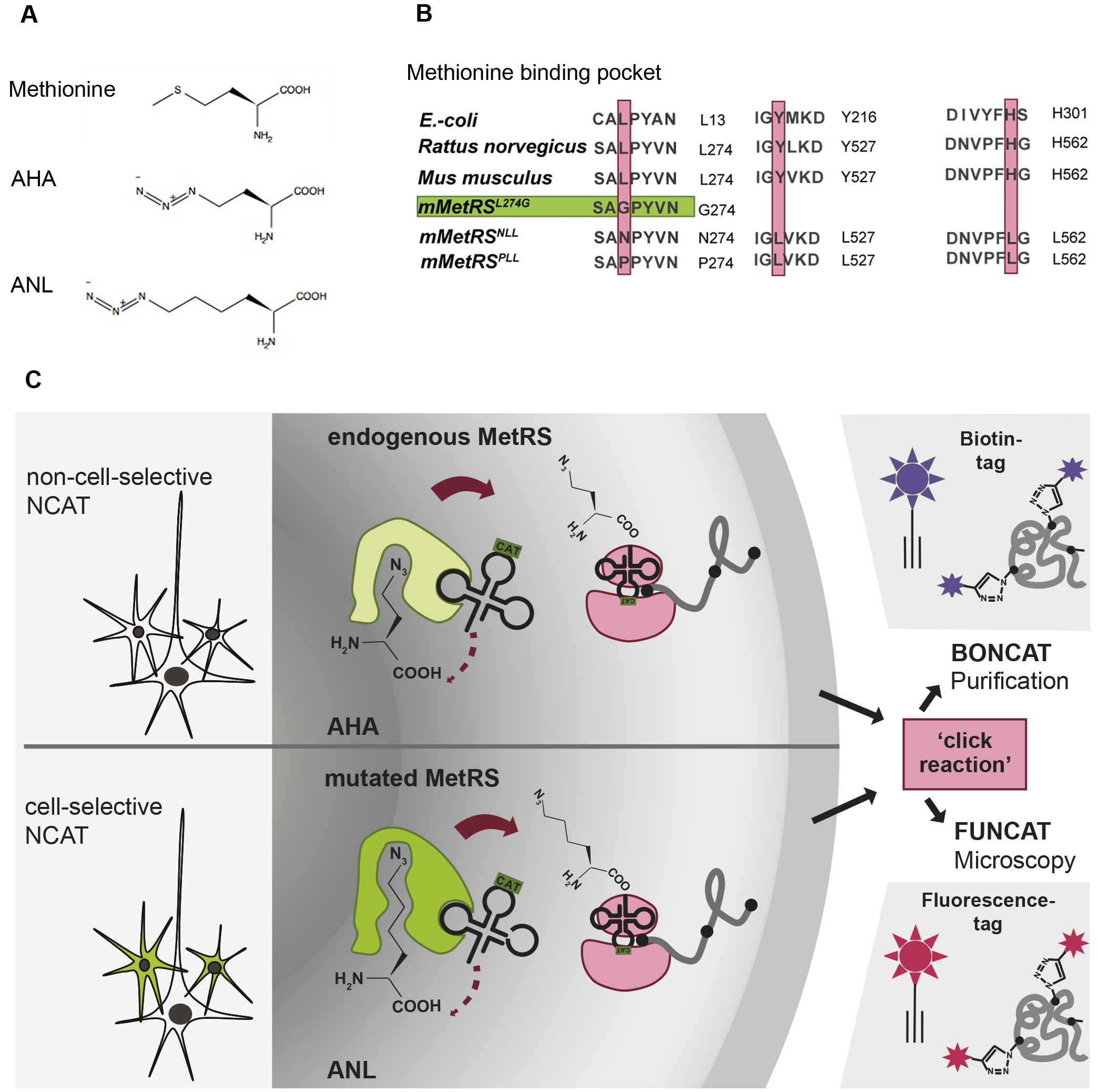

Supplement: S1 Fig — (A) Whereas AHA is sterically similar to methionine and can be processes by the endogenous methionyl-tRNA synthetase, the side chain of ANL is longer and, therefore, excluded by the wild type enzyme. (B) The Met binding pocket is highly conserved and sequences are comparable between the Escherichia coli and Mus musculus. Amino acids that substantially shape the binding pocket are highlighted in pink. To allow for binding of the long-chained ANL, three Mus musculus MetRS (mMetRS) mutants were generated, one being created by a single amino acid exchange at position 274 leading to mMetRSL274G (highlighted in green). Two more combinations of amino acid exchanges led to mMetRSPLL and mMetRSNLL. (C) Labeling of de novo synthesized proteins can be done in a non-cell-selective fashion with bioorthogonal, non-canonical amino acids such as the azide-bearing azidohomoalanine (AHA). AHA is utilized by the endogenous methionyl-tRNA synthetase (MetRS) and replaces methionine (Met) during the course of protein translation. Due to the azide-harboring side chain of AHA, AHA-carrying proteins can be visualized by covalent reaction to a fluorescent-alkyne tag (FUNCAT) or purified via a biotin-alkyne tag (BONCAT) using ‘click reaction'. Processing of azidonorleucine (ANL) affords a mutated form of the MetRS with an enlarged Met binding pocket that allows ANL to be coupled to the respective tRNAMet. When cells are genetically manipulated to express the mutated MetRS, cell type-specific ANL processing and integration of ANL into newly synthesized proteins becomes feasible. ANL labeling in astrocytes can be achieved with expression of the mutated MetRS under a promoter for specific astrocytic proteins like GFAP, respectively. Also here, the terminal azide group within ANL allows visualization or purification of labeled proteins following 'click reaction'. (TIF) [file pone.0145451.s001.tif]

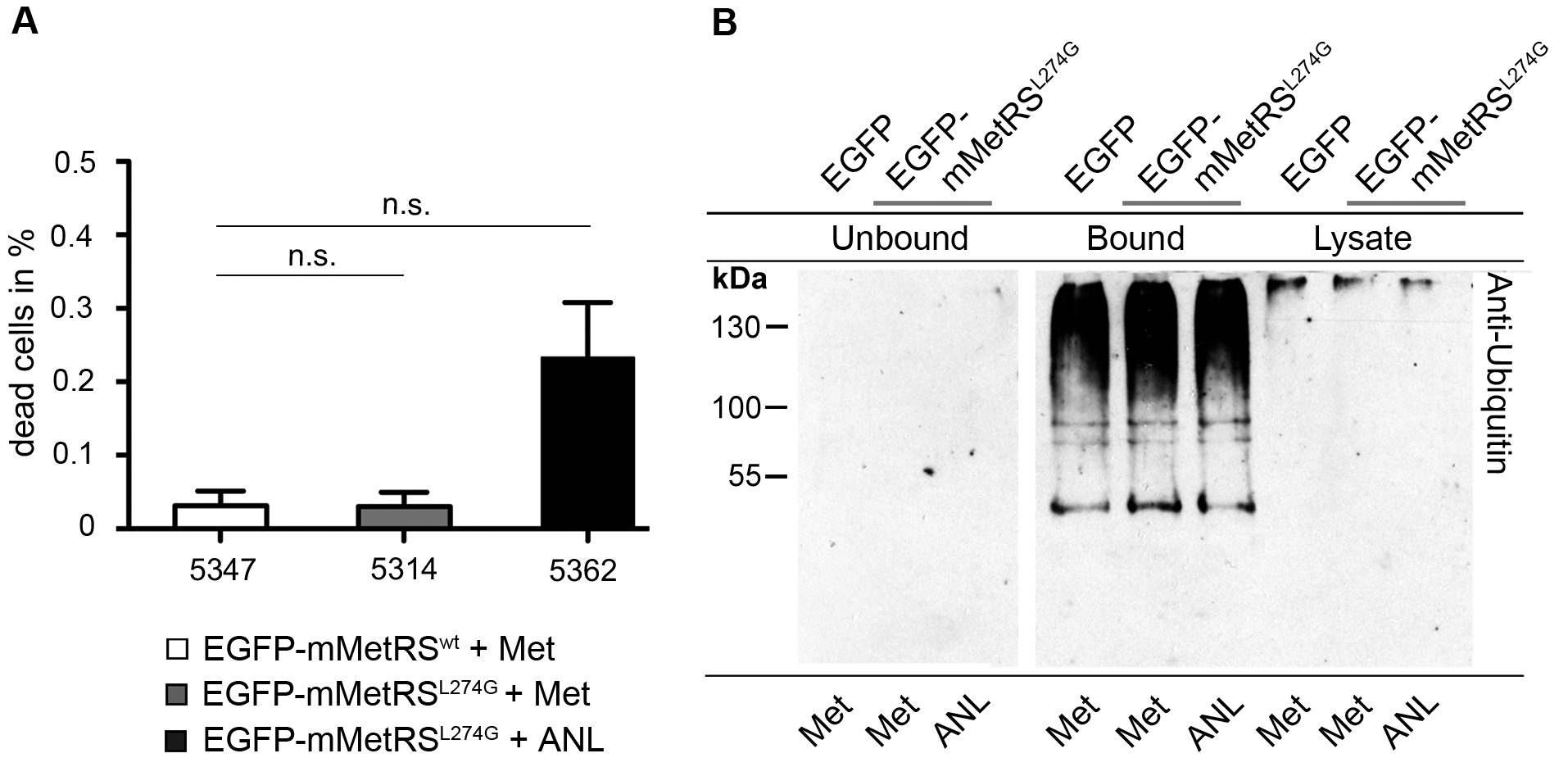

Supplement: S2 Fig — (A) HEK293T cells were transfected with either the EGFP-mMetRSwt or the mutant enzyme version EGFP-mMetRSL274G and incubated for 4 h with 4 mM ANL or Met in Met-free medium. A live-staining with a 2 μg/ml propidium iodide solution revealed a slight tendency towards an increase of dead cells with propidium iodide positive nuclei in cells expressing EGFP-mMetRSL274G and ANL treatment compared to Met or EGFP-mMetRSwt control in HEK293T cells. The percentage of dead cells refers to the total number of counted cells as indicated at the X-axis (represented data are mean +/- SEM; n = 5 independent experiments; One-way Anova, p>0.05). (B) HEK293T cells overexpressing EGFP or EGFP-mMetRSL274G were incubated with either 4 mM Met or ANL in Met-free medium for 2 h. Cell lysates underwent immunoprecipitation applying an anti-Ubiquitin antibody to pull down ubiquitinated proteins. No changes of ubiquitinated protein levels in cells that overexpressed EGFP-mMetRSL274G and that are incubated with ANL are revealed when compared to Met-treated controls. Both images originate from the same blot. (TIF) [file pone.0145451.s002.tif]

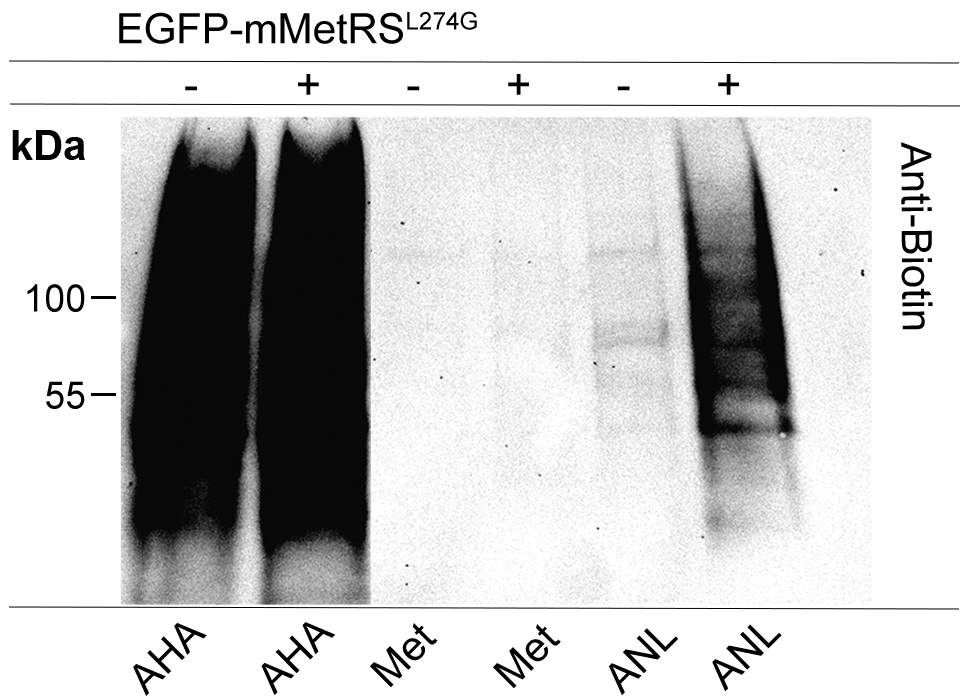

Supplement: S3 Fig — Primary neuron-glia cocultures (DIV 21), either infected with LVGFAPEGFP-mMetRSL274G or not treated, were incubated with either 4 mM AHA, ANL or Met for 4 h. Successful ANL integration into proteins was detected with a biotin-alkyne tag using ‘click-reaction’ of cell lysates, followed by immunodetection of biotinylated proteins. Also here, ANL integration is specific for cultures expressing EGFP-mMetRSL274G only. (TIF) [file pone.0145451.s003.tif]

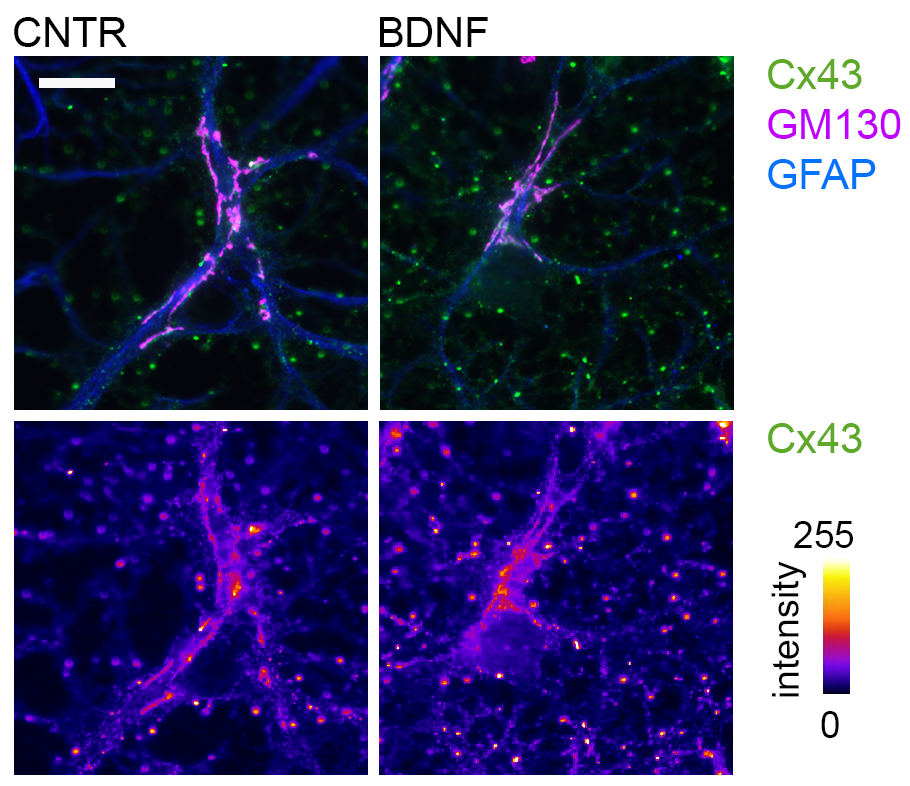

Supplement: S4 Fig — Neuron-glia cocultures (DIV 22) were infected with LVGFAPEGFP and treated with 50 ng/ml BDNF for 4 h. Cx43 positive signals were observed in the Golgi apparatus of astrocytes applying immunocytochemistry for Cx43 and the Golgi marker GM130. Cx43 signal intensities are color coded in the lower panel. No increase of de novo synthesized Cx43 within the Golgi apparatus after BDNF application was observed (scale bar = 10 μm). (TIF) [file pone.0145451.s004.tif]

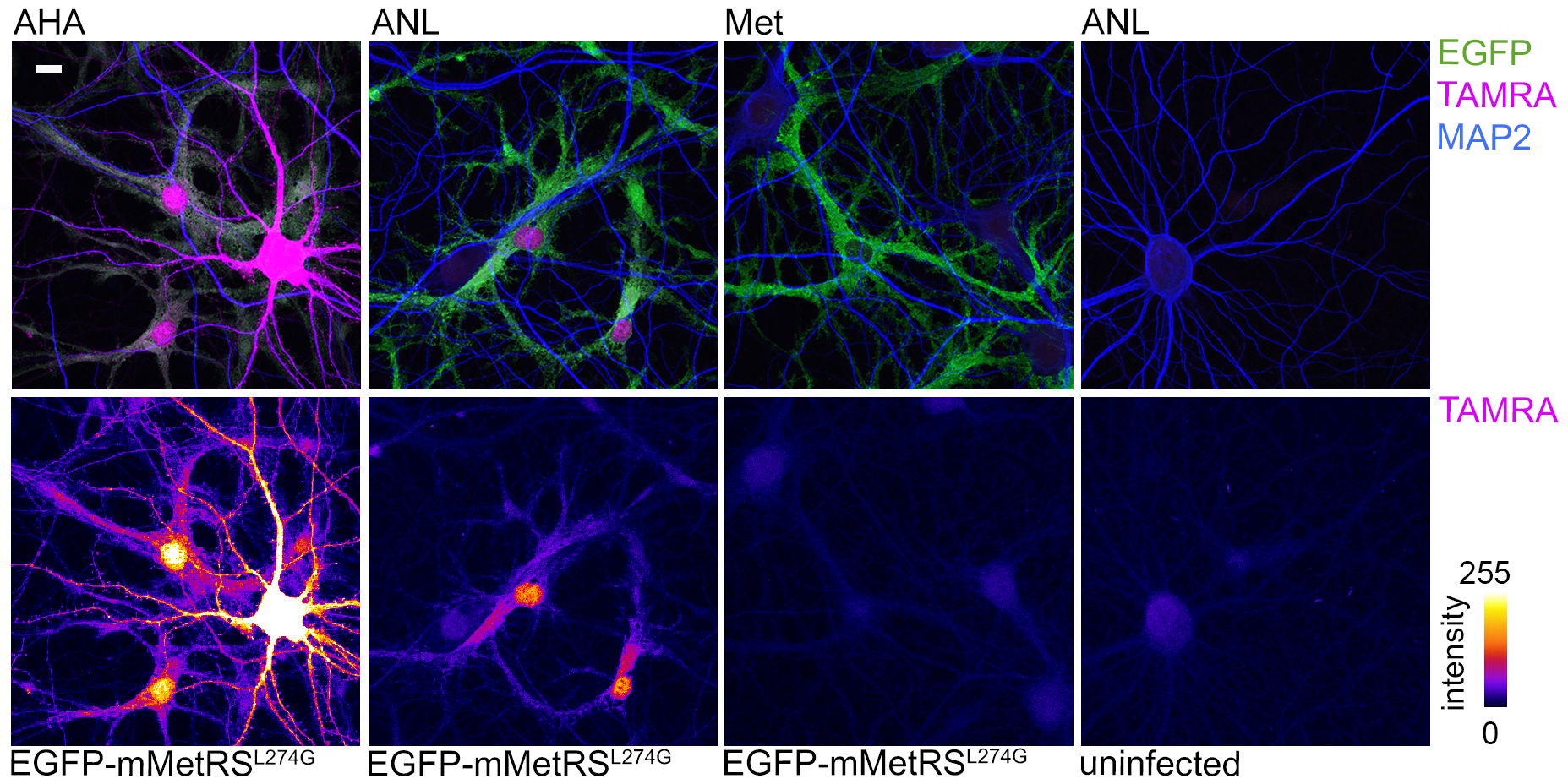

Supplement: S5 Fig — Primary neuron-glia cocultures (DIV 22), infected with the lentivirus LVGFAPEGFP-mMetRSL274G, expressed the enzyme cell type-specific in astrocytes. Cells were incubated with either 4 mM AHA, ANL or Met for 4 h in Met-free medium and AHA or ANL incorporation was visualized by a TAMRA-alkyne tag. AHA labeling occurred in a non-cell-selective manner both in neurons and astrocytes. TAMRA-positive proteins are detected solely in EGFP-mMetRSL274G expressing astrocytes when labeled with ANL. MAP2 positive neurons integrate AHA into de novo synthesized proteins whereas only background staining is found in neurons incubated with ANL or Met (scale bar = 10 μm) (TIF) [file pone.0145451.s005.tif]

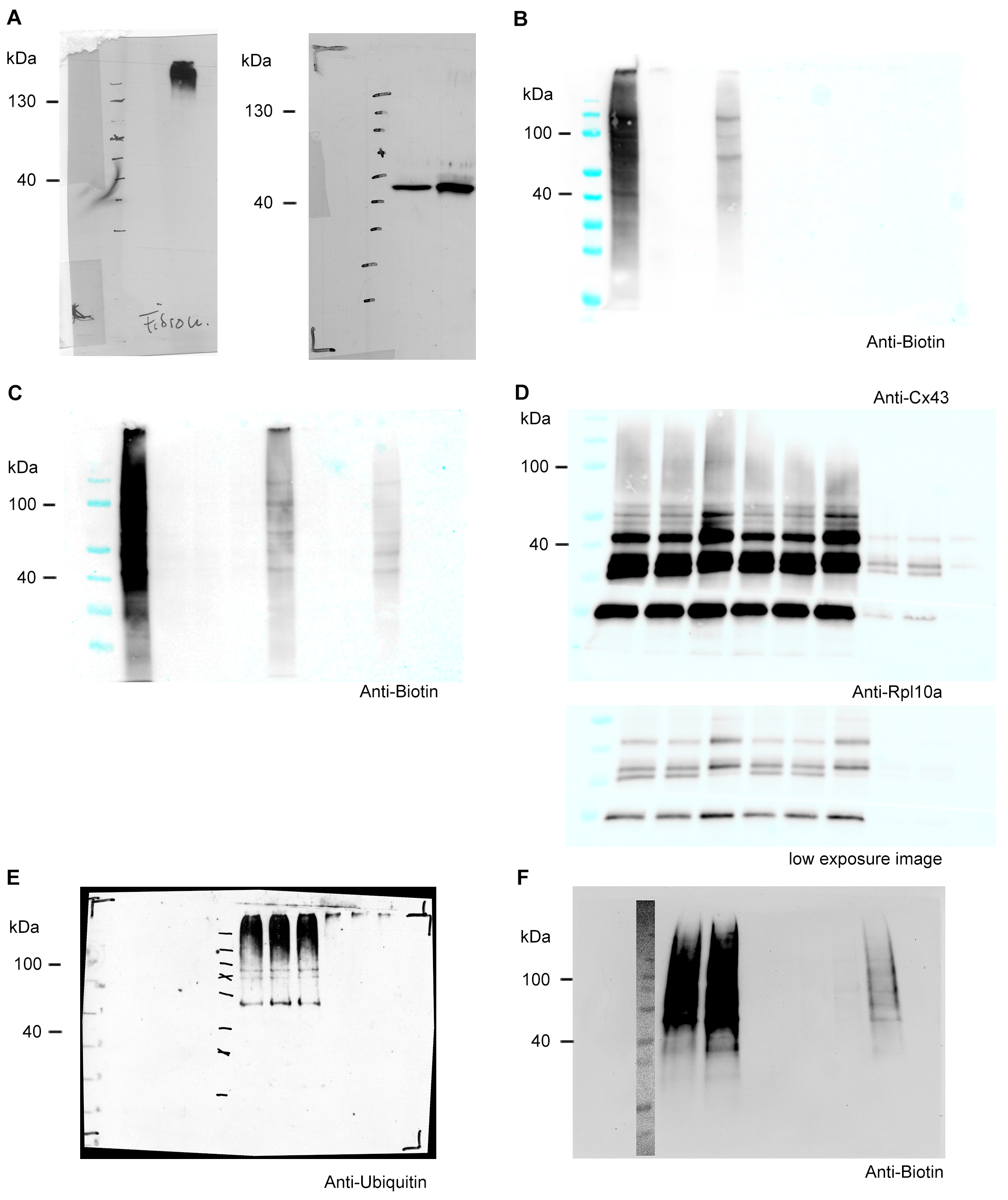

Supplement: S6 Fig — (A) Original immunoblots related to Fig 1B. (B) Original immunoblots related to Fig 3B. The layer with the protein ladder was omitted in the main figure. (C) Original immunoblots related to Fig 3D. The layer with the protein ladder was omitted in the main figure. (D) Original immunoblots related to Fig 6A. The layer with the protein ladder was omitted in the main figure. The lower image represents the same blot with lower exposure to display both input and supernatant fractions that are enhanced in the main figure. (E) Original immunoblots related to S2 Fig. (F) Original immunoblots related to S3 Fig. (TIF) [file pone.0145451.s006.tif]
