# Supplementary material for: Monitoring Astrocytic Proteome Dynamics by Cell Type-Specific Protein Labeling
Source: PLoS One. 2015 Dec 21;10(12):e0145451. doi: 10.1371/journal.pone.0145451 (PMC4686566; doi:10.1371/journal.pone.0145451)
Supplement: S1 File — (DOC) [file pone.0145451.s007.doc]

## S1 Supplemental Material and Methods

### Preparation of Azidohomoalanine and Azidonorleucine

Azidohomoalanine (AHA) was prepared as described previously [1]. Azidonorleucine was prepared as described previously for azidohomoalanine via copper-catalyzed diazo transfer [1]. Briefly, *N*_α_-(*tert*-Butoxycarbonyl)-L-lysine was converted to Boc-protected ANL using the azidification reagent triflic acid. The free amino acid was obtained upon treatment with acid and was purified by ion-exchange chromatography. The biotin-alkyne tag (biotin-PEO-propargylamine) was synthesized as described previously [2].

### **Generation of mMetRS with mutated Met-binding pockets**

According to previously published *Escherichia coli* MetRS mutants we generated the following *Mus musculus* mutants with an enlargement of the methionine binding pocket: mMetRS^L274G^: L274G; mMetRS^NLL^: L274N, Y527L, H562L; mMetRS^PLL^: L274P, Y527L, H562L [3,4]. Cloning of *Mus musculus* MetRS^wt^ fused to EGFP was done by PCR with primers MetRSfwd and MetRSrev (see table S1) using I.M.A.G.E. clone 6416029 (GenBank ID BC079643, ATCC) as a PCR template. The resulting PCR product was cloned into pEGFP-C1 vector (Clontech) using EcoRI restriction sites. The construct EGFP-mMetRS^wt^ was used as a template for the generation of all modified MetRS constructs. The introduction of point mutations to generate the binding pocket mutants EGFP-mMetRS^L274G^, EGFP-mMetRS^NLL^, EGFP-mMetRS^PLL^ was done by inner primer mutagenesis with two primers for each mutation site in combination with the respective forward or reverse MetRS primers as listed in the table below. The PCR products were used as a template in an additional PCR reaction to generate the full-length sequence of the corresponding mutated MetRS using primers MetRSfwd and MetRSrev. These two flanking primers allow the insertion of DNA fragments into the pEGFP-C1 vector after restriction with EcoRI.

The EGFP-mMetRS^L274G^ construct was used to generate a lentiviral expression vector. First, an internal BamHI restriction site was removed by an introduction of a silent mutation via inner primer mutagenesis with the primer pair MetRS R2106Rfwd and MetRS R2106Rrev in combination with primers MetRSfwd and MetRSLVrev (see table below). The full-length construct was generated as described above and used for all lentiviral expression constructs generated in this study.

The FUGW lentiviral expression vector was generated as follows: For expression of mMetRS^L274G^ in glia cells and neurons, additional restriction sites NheI and BamHI were introduced into the lentiviral FUGW vector by primer annealing and insertion into the BamHI and EcoRI restriction sites downstream of the promoter using the primers LVlinkerfwd and LVlinkerEcoRIrev as listed below [5]. EGFP-mMetRS^L274G^ was subcloned from pEGFP-C1 by NheI and BamHI restriction. For cell type-specific expression of EGFP-mMetRS^L274G^ or EGFP alone in GFAP positive astrocytes the ubiquitin promoter of FUGW was removed by PstI restriction. The HIV-1 flap segment was reinserted by PCR with FUGW as a template and primers FUGWPromfwd and FUGWPromrev (Table 1).

### Table 1. Primer Sequences

| **Construct** | **Primer name** | **Primer sequence** |
| --- | --- | --- |
| MetRS^wt^ | MetRSfwd | 5'- tcgaattcggcacgaggccgaggc-3' |
|  | MetRSrev | 5'- gcagaattcgcccttgcgcgaaat -3' |
| MetRS^L274G^ | L274GIPMfwd:  L274G | 5-'tcaccagtgccggcccctatgtca-3 |
|  | L274GIPMrev:  L274G | 5’-tgacataggggccggcactggtga-3’ |
| MetRS^NLL^ | NLLIPMfwd:  L274N  Y527L  H562L | 5'- tcaccactgccaacccctatgtca-3  5'- tactattggcttggtgtccatca-3'  5’- at gttcccttccttggcttggtc-3’ |
|  | NLLIPMrev:  L274N  Y527L  H562L | 5'-tgacataggggttggcactggtga-3'  5'- tgatggacaccaagccaatagta-3'  5’- gaccaagccaaggaagggaacat-3’ |
| MetRS^PLL^ | PLLIPMfwd:  L274P | 5’- tcaccagtgccccaccctatgtca-3’ |
|  | PLLIPMrev:  L274P | 5'- tgacatagggtggggcactggtga-3' |
| MetRS-BamHI | MetRSR2106Rfwd | 5'- gaaaaggttcgaatccgggatgcc -3' |
|  | MetRSR2106Rrev | 5'- ggcatcccggattcgaaccttttc -3' |
|  | MetRSLVrev | 5'- gtcgaattcatcactttttcttcttgcc -3' |
| FUGW Promoter | FUGWPromfwd | 5’- ccaattctgcagacaaatggcagt -3’ |
|  | FUGWPromrev | 5’- catctgcagatctccttaattaaccaaact -3’ |
| LV linker | LV linkerfwd | 5'- gatcggctagcatggatcctagataactgac -3’ |
|  | LV linkerrev | 5'- gatcgtcagttatctaggatccatgctagcc -3' |
|  | LV linkerEcoRIrev | 5’- aattgtcagttatctaggatccatgctagcc -3’ |

A modified form of the GFAP promoter version gfaABC_1_D [6] was reinserted into this modified FUGW by restriction digestion using EcoRI and BglII and the subsequent ligation into the EcoRI and BamHI restriction sites of the FUGW expression vector. Additional restriction sites NheI and BamHI were introduced downstream of the GFAP promoter for subcloning of mMetRS^L274G^ or of EGFP alone by inner primer annealing and insertion as described above.

### Lentivirus preparation

The preparation of lentiviruses was done as previously published but instead of Δ8.9 the packaging vector psPAX2 (Trono Lab, unpublished; Addgene #12260) was used [5]. The virus titer was determined in neuron-glia cocultures via immunocytochemistry staining with an antibody against GFP as described in material and methods.

### Cell culture and transfection

HEK293T cells (DSMZ, ACC 635) were maintained in Dulbecco's modified eagle medium (DMEM) containing 10% fetal bovine serum, 2 mM glutamine, antibiotics and antimycotics (100 U/ml penicillin, 100 µg/ml streptomycin, 250 ng/ml Fungizone®, all from Invitrogen) at 37°C and 5% CO_2_. Transient transfection of HEK293T cells was done using the calcium phosphate method. Briefly, DNA was dissolved in 500 mM CaCl_2_ buffer and added to 140 mM NaCl, 50 mM HEPES, 1.5 mM Na_2_HPO_4_, (pH 7.05) buffer. After 1 min incubation the mixture was added dropwise to the cells and the medium was exchanged against fresh culture medium after 4 h. Cells were cultured for additional 24 h and either fixed with 4% paraformaldehyde (Roth) in 1x PBS for immunocytochemistry or collected from culture plates in cold 1x PBS (pH 7.4) for immunoprecipitation assays.

Cortical neuron-glia cocultures were prepared from cortices of E18–E19 Wistar rats as described previously [7]. Cells were plated on poly-D-lysine coated coverslips at densities specified (20000 cells/well in 24-well plate and 3 million cells per 75 cm^2^ flask) and kept in DMEM including 10% fetal bovine serum, 2 mM glutamine and antibiotics (100 U/ml penicillin, 100 µg/ml streptomycin, all from Invitrogen) at 37°C and 5% CO_2_. The medium was replaced after 24 h by Neurobasal medium, supplemented with 1x B27 and 0.8 mM glutamine (all from Invitrogen). Cells were fed once a week with 10% fresh medium. After a washing step with 1x HBSS (Invitrogen) or 1x PBS (pH 7.4) supplemented with 0.1 mM CaCl_2_ and 1 mM MgCl_2_ cells were either fixed for immunocytochemistry as described above unless noted otherwise or scraped from flasks in cold 1x PBS (pH 7.4) or 1x PBS (pH 7.8) and pelleted at 2000 g for 5 min at 4°C. Astrocytic monocultures were prepared from P2–P3 Wistar rats analogous to neuronal cultures. Cells were cultured in DMEM including 10% fetal bovine serum, 2 mM glutamine and antibiotics (100 U/ml penicillin, 100 µg/ml streptomycin, all from Invitrogen) at 37°C and 5% CO_2_. The medium was replaced after 24 h and thereupon every 3–4 days. Prior to use, microglia were removed by shaking and washing steps with 1x HBSS (Invitrogen). Astrocytes were detached with TrypLE^TM^ (Invitrogen) and plated on glass cover slips.

### SDS-PAGE and Immunoblotting

Samples for immunoblotting were solubilized in 4x sample buffer containing 250 mM Tris-HCl (pH 6.8), 1% SDS, 40% glycerol, 20% ß-mercaptoethanol, 0.004% bromophenol blue, incubated at 95°C for 5 min, subsequently separated on one-dimensional SDS-PAGE using Tris-based 9.5% polyacrylamide gels and transferred to 0.45 µm nitrocellulose membranes (GE Healthcare). Membranes were incubated for 10 min in 3% acetic acid containing 0.5% Ponceau S (Roth) and then incubated in blocking buffer containing 5% dry milk and 0.1% Tween® 20 (Roth) in 1x Tris buffered saline (1x TBS) for 1 h. Membranes were subsequently incubated with the primary antibodies dissolved in 1x TBS containing 0.1% Tween® 20 (Roth) except for anti-Biotin antibodies that were incubated in blocking buffer ON at 4°C. After a series of washing steps the membrane was incubated with peroxidase-coupled antibodies in blocking buffer for 90 min at RT and further developed with ECL reagent (Thermo Fisher Scientific) using Amersham Hyperfilm^TM^ ECL (GE Healthcare), the ChemoCam Imager (Intas) or the Odyssey® Fc luminescence detector (LI-COR).

To control for equal protein loading the samples were separated by SDS-PAGE and stained for 1 h with 0.05% Coomassie brilliant blue dissolved in 50% methanol and 10% acetic acid and destained using destaining solution (5% methanol and 7% acetic acid).

### Metabolic labeling with noncanonical amino acids in HEK293T and Neuron-glia cocultures

For integration of azidohomoalanine (AHA), azidonorleucine (ANL) or methionine (Met) into newly synthesized proteins, cells were washed with pre-warmed (37°C) 1x HBSS. HEK293T cells were pre-incubated for 20 min in Met- and cysteine-free DMEM (Invitrogen), supplemented with 0.2 mM L-cysteine, 10% fetal bovine serum, 2 mM glutamine, antimycotics (100 U/ml penicillin, 100 µg/ml streptomycin, 250 ng/ml Fungizone®, all from Invitrogen) before incubation with ANL, AHA or methionine (all 4 mM) for indicated labeling durations.

For the detection of newly synthesized proteins via FUNCAT, HEK293T cells were transfected with EGFP-mMetRS^wt^ or EGFP-mMetRS^L274G^. On the following day, cells were incubated with either 4 mM AHA or ANL with or without 40 µM anisomycin for indicated time periods. For the analysis of ANL integration via immunoblotting HEK293T cells overexpressing either EGFP-mMetRS^wt^, EGFP-mMetRS^NLL^, EGFP-mMetRS^L274G^ or EGFP-mMetRS^PLL^ were incubated with 4 mM ANL, AHA or Met in Met-free DMEM with or without 40 µM anisomycin and 100 µg/ml cycloheximide for incubation times indicated. Neuron-glia cocultures were pre-incubated in Hibernate medium lacking Met, supplemented with 0.8 mM glutamine, 1x B27, penicilin (100 U/ml), streptomycin (100 µg/ml; all from Invitrogen), for 20 min and then incubated with either 4 mM AHA, ANL or Met for indicated time periods [8]. After incubation cells were washed with phosphate buffered saline (PBS; pH 7.4) supplemented with 1 mM MgCl_2_ and 0.1 mM CaCl_2_ and either fixed with 4% paraformaldehyde (Roth) for FUNCAT analyzes or collected in 1x PBS (pH 7.8) for BONCAT reaction.

### Immunoprecipitation of ubiquitinated proteins

HEK293T cells that were either transfected with EGFP-mMetRS^L274G^ or EGFP as a control (both based on pEGFP-C1, Clontech) were pre-incubated in Met-free medium and subsequently labeled with ANL or Met for 2 h as described in material and methods. Cell pellets were homogenized in 20 mM Tris-HCl (pH 7.4), 150 mM NaCl, 2x *cOmplete* EDTA-free Proteinase Inhibitor (Roche), 100 nM MG-132 (Calbiochem) and supplemented with 1% Triton^TM^ X-100 (Roth). Equal protein amounts were pre-cleaned with Protein A/G Plus agarose (Santa Cruz) and then incubated with the mouse monoclonal antibody against Ubiquitin (FK2, Assay Designs) for 1 h. Additional application of agarose and ON incubation at 4°C was followed by a series of washing steps in the following order: two times each 20 min with buffer 1 containing 50 mM Tris, 0.15 M NaCl, 1 mM EDTA, 0.1% Igepal CA-630 (pH 7.5), once with buffer 2 comprised of 50 mM Tris, 0.15 M NaCl, 0.1% Igepal CA-630 (pH 7.5) and one time with 50 mM Tris 0.1% Igepal CA-630, (pH 7.5). Bound proteins were eluted by boiling the samples at 95°C for 5 min in 4x sample buffer containing 250 mM Tris, 20% ß-mercaptoethanol, 1% SDS, 40% glycerol, 0.004% bromophenolblue (pH 6.8). Ubiquitinylated proteins were separated by SDS-PAGE and analyzed by immunoblotting as described above.

### Propidium Iodide Staining

HEK293T cells grown on glass coverslips in standard cell culture medium were transfected with either EGFP-mMetRS^wt^ or EGFP-mMetRS^L274G^ (both based on pEGFP-C1, Clontech) as described above. On the following day cells were incubated with either 4 mM ANL or 4 mM Met for 4 h in Met-free medium as described above. The medium was exchanged to 2 µg/ml propidium iodide in 1x PBS (pH 7.4) and incubated for 5 min, then cells were washed with 1x PBS (pH 7.4) and fixed with 4% PFA in 1x PBS (pH 7.4). Coverslips were mounted and propidium iodide positive stained cells expressing EGFP-mMetRS^wt^ or EGFP-mMetRS^L274G^ were counted immediately after mounting. For a positive control propidium iodide staining was done after the 4% PFA fixation step.

Propidium iodide staining of neuron-glia cocultures infected with LVGFAPEGFP-mMetRS^L274G^ and labeled with Met, AHA, or ANL for 4 h was performed as described for HEK293T cells.

## Suppl**emental References**

1. Link AJ, Vink MKS, Tirrell DA. Preparation of the functionalizable methionine surrogate azidohomoalanine via copper-catalyzed diazo transfer. Nat Protoc. 2007;2: 1879–1883. doi:10.1038/nprot.2007.268

2. Link AJ, Tirrell DA. Cell surface labeling of Escherichia coli via copper(I)-catalyzed [3+2] cycloaddition. J Am Chem Soc. 2003;125: 11164–11165. doi:10.1021/ja036765z

3. Link AJ, Vink MKS, Agard NJ, Prescher JA, Bertozzi CR, Tirrell DA. Discovery of aminoacyl-tRNA synthetase activity through cell-surface display of noncanonical amino acids. Proc Natl Acad Sci U S A. 2006;103: 10180–10185. doi:10.1073/pnas.0601167103

4. Tanrikulu IC, Schmitt E, Mechulam Y, Goddard WA, Tirrell DA. Discovery of Escherichia coli methionyl-tRNA synthetase mutants for efficient labeling of proteins with azidonorleucine in vivo. Proc Natl Acad Sci U S A. 2009;106: 15285–15290. doi:10.1073/pnas.0905735106

5. Lois C, Hong EJ, Pease S, Brown EJ, Baltimore D. Germline transmission and tissue-specific expression of transgenes delivered by lentiviral vectors. Science. 2002;295: 868–872. doi:10.1126/science.1067081

6. Lee Y, Messing A, Su M, Brenner M. GFAP promoter elements required for region-specific and astrocyte-specific expression. Glia. 2008;56: 481–493. doi:10.1002/glia.20622

7. Banker G, Goslin K. Developments in neuronal cell culture. Nature. 1988;336: 185–186. doi:10.1038/336185a0

8. Brewer GJ, Price PJ. Viable cultured neurons in ambient carbon dioxide and hibernation storage for a month. Neuroreport. 1996;7: 1509–1512.
